# Investigation of Potential Risks in Bio‐Based Food Contact Materials (FCMs) From Microbial Exploitation of Agricultural Wastes: Case Studies of Bacterial Cellulose and Nisin

**DOI:** 10.1111/1541-4337.70480

**Published:** 2026-04-09

**Authors:** Marianna Ciccone, Emmanouil Tsochatzis, Joel Armando Njieukam, Lorenzo Siroli, Davide Gottardi, Rosalba Lanciotti, Francesca Patrignani

**Affiliations:** ^1^ Department of Agricultural and Food Sciences, Campus of Food Science, Alma Mater Studiorum University of Bologna Cesena Italy; ^2^ Centre for Innovative Food (CiFOOD), Department of Food Science Aarhus University Aarhus Denmark; ^3^ Interdepartmental Centre for Agri‐Food Industrial Research, Campus of Food Science, Alma Mater Studiorum University of Bologna Cesena Italy

**Keywords:** bacterial cellulose, circular economy, food by‐products, food contact materials, nisin, safety assessment

## Abstract

In the context of the circular economy and the increasing demand for safe and sustainable packaging, this work addresses the safety assessment of food contact materials (FCMs) derived from agro‐industrial by‐products. Despite growing interest in these bio‐based materials, the literature still lacks a structured safety‐assessment framework able to account for substrate‐related contaminants, microbial processing, and downstream impurities. The novelty of this work lies in applying the European Food Safety Authority (EFSA) Technical Report perspective on natural mixtures to two representative case studies: bacterial cellulose (BC) produced by *Komagataeibacter* spp., as a promising microbial biopolymer for food packaging application, and nisin produced by *Lactococcus lactis* subsp. *lactis*, as an antimicrobial peptide, to functionalize the packaging material. This study aims to evaluate whether the EFSA‐oriented framework can support the identification of potential substances of concern across the production chain when cellulose is produced starting from agro‐industrial waste or nisin is applied. For this, a systematic literature review (SLR) was conducted to investigate potential substances of concern from agro‐industrial substrates through fermentation to the final activated materials. The findings highlight the need to characterize natural carbon sources, including pesticide residues, consider the qualified presumption of safety (QPS) status of production microorganisms, and assess metabolites and fermentation by‐products. The behavior of these substances during processing and their potential migration into food are critical aspects. A preliminary safety assessment at early development stages is therefore essential to guide material design and regulatory compliance. Overall, this study provides a practical framework to support researchers, developers, and risk assessors in identifying safety concerns and improving the regulatory readiness of innovative bio‐based FCMs.

## 1 Introduction

In 2023, 79.7 million tons of packaging waste were generated in the EU. The largest share of this was made up of paper and cardboard, amounting to 32.3 million tons. This corresponded to 40.4% of the total. There were also substantial amounts of plastic packaging waste, at 15.8 million tons (19.8%), glass at 15.0 million tons (18.8%), and wood at 12.6 million tons (15.8%; Eurostat 2023). The rise of single‐use packaging has strained the waste management system, complicating recycling due to the complexity of materials and infrastructure limitations. This issue has been exacerbated by the growing popularity of food delivery services during the Coronavirus disease 2019 pandemic (COVID‐19), which contributed to an increase in single‐use plastics, including complex or multilayer materials that are often difficult to recycle due to material composition or contamination with food residues (European Commission Joint Research Centre 2024; Sharma et al. 2020). Nonbiodegradable polymers pose significant environmental risks due to their limited recyclability and persistence in ecosystems (Sharma et al. 2025). To address these issues, the European Union launched the “European Green Deal” in 2019, prioritizing circular economy strategies (Fetting 2020; Olczyk and Kuc‐Czarnecka 2025). A key focus is reducing single‐use packaging and promoting reusable alternatives, such as reusable container systems, deposit‐return schemes, and refill‐based packaging models, to mitigate the environmental and health impact of plastic waste. However, the sustainability of such solutions depends on factors such as the actual number of reuse cycles, collection efficiency, and resource consumption for cleaning, sanitization, production, and transportation (Sinha 2024).

Beyond packaging, agricultural and food waste represent major global challenges, both in terms of resource inefficiency and environmental impact. Recent estimates indicate that approximately one‐third of all food produced globally is lost or wasted, corresponding to nearly 1.3 billion tons per year (Dey et al. 2025; Kilemile et al. 2025). This issue is expected to intensify, as global food production will need to increase significantly by 2050 to meet the demands of a projected population of 9.7 billion people.

Reducing agricultural and food waste is therefore a key component of circular economy strategies, promoting the recovery and valorization of valuable compounds and contributing to more sustainable resource use. In this context, technological advancements have enabled the conversion of agri‐food by‐products into value‐added materials (Boudalia et al. 2026; Ligarda‐Samanez et al. 2025). These materials can be directly extracted from biomass or synthesized through microbial fermentation using agri‐food residues as carbon sources, opening new opportunities for sustainable food packaging applications (Brugnoli et al. 2023a; Mehdizadeh et al. 2026).

Such processes can generate a wide range of products, including biopolymers, bioactive compounds, organic acids, and antimicrobial peptides, which have applications across food, packaging, pharmaceutical, cosmetic, and feed sectors (Adeyeye and Sankarganesh 2026; Brugnoli et al. 2023b; Gottardi et al. 2022, 2023; Oyewole et al. 2026; Rossi et al. 2024; Siroli et al. 2022; Younis et al. 2026; Zotta et al. 2020).

However, the large‐scale implementation of these processes remains challenging. Variability in waste composition, seasonal availability of raw materials, and the need for consistent process control can affect both technical feasibility and economic viability. In this regard, advances in biotechnology have enabled the controlled production of biopolymers such as alginates, bacterial cellulose (BC), and gellans, allowing improved control over material properties and functionality (Brugnoli et al. 2023b; Gullo et al. 2018; Panda et al. 2024; Sharma et al. 2026; Younis et al. 2026).

An innovative natural polymeric material gaining special interest in the food industry and food packaging is BC, that is, cellulose produced from microbial fermentation. Recent studies have shown that the composition and interaction of microbial communities involved in fermentation processes, including yeasts, acetic acid bacteria, and lactic acid bacteria, can significantly influence metabolite production and process efficiency, thereby affecting the properties and yield of bio‐based materials (Lasagni et al. 2024; Njieukam et al. 2024; Signorello et al. 2026). BC offers good mechanical, physical, and barrier properties, making it highly suitable for food packaging. Its unique structural characteristics provide an effective moisture and oxygen barrier, enhancing packaging performance. Additionally, BC can incorporate and release antimicrobial agents, prolonging their activity and improving food preservation. Beyond antimicrobial applications, BC can also be functionalized with inorganic compounds or nanoparticles to tailor its physicochemical properties, further expanding its potential applications (Barbi et al. 2025; Haghighi et al. 2021; Xing et al. 2026; Zhao et al. 2026). Such active packaging helps in extending the shelf life and improving the nutritional and sensory properties of food by preventing or inhibiting the growth of pathogenic and spoilage microorganisms, ensuring food safety throughout the distribution chain (Patrignani et al. 2016; Siroli et al. 2017, 2023). Bacteriocins such as nisin have gained attention due to their strong inhibitory effect against pathogenic microorganisms. Nisin has been widely used for over six decades as a food preservative, retaining its stability and activity even after pasteurization and sterilization, which makes it particularly suitable for integration into polymer‐based antimicrobial packaging (Bukvicki et al. 2023; Gottardi et al. 2021; Siroli et al. 2019, 2020).

Bio‐based food contact materials (FCMs) and articles are EU‐regulated to ensure their food safety. The framework Regulation (EC) No 1935/2004 (Regulation (EC) No 1935/2004) requires that materials do not transfer their constituents to food in quantities that could endanger human health. Additional EU measures (e.g., for plastics and active materials) may also apply to bio‐based materials such as BC and nisin. These materials are subject to European Food Safety Authority (EFSA) evaluation prior to authorization by the European Commission (EC), and data must be submitted in accordance with EFSA guidance (European Food Safety Authority (EFSA) 2021). Moreover, since bio‐based materials derived from agri‐food by‐products are often complex mixtures with variable and partially uncharacterized composition, EFSA published in 2023 a Technical Report outlining principles for the safety assessment of mixtures of natural origin used in FCM production (European Food Safety Authority (EFSA) et al. 2023). These principles are therefore relevant for the safety assessment of such materials.

Despite the increasing interest in bio‐based FCMs obtained from agro‐industrial by‐products, the current literature predominantly focuses on material performance, functionality, and sustainability aspects, while the safety assessment of these materials remains insufficiently structured. Limited attention has been given to the systematic identification of potential substances of concern across the entire production chain, including substrate‐derived contaminants, microbial metabolism, and downstream processing residues. In this context, the present study addresses this gap by applying the principles outlined in the EFSA Technical Report on natural mixtures to two representative case studies: BC produced by *Komagataeibacter* spp. and nisin produced by *Lactococcus lactis* subsp. *lactis*. The novelty of this work lies in combining an EFSA‐oriented safety perspective with a systematic literature review (SLR) to map potential hazards from agro‐industrial substrates to the final FCM. This approach aims to evaluate whether such a framework can support the early identification of safety‐relevant issues and contribute to the safer‐by‐design development of innovative bio‐based FCMs.

More specifically, the study considers three main stages of the production chain: (i) the composition and potential contamination of agro‐industrial by‐product substrates, (ii) the microbial fermentation process, including the identity and qualified presumption of safety (QPS) status of the production organisms and the formation of metabolites, and (iii) downstream processing and recovery, with particular attention to residual substances and their potential migration into food. The applicability of the EFSA Technical Report approach is evaluated based on its ability to systematically identify relevant categories of substances of concern across these stages, to highlight key knowledge gaps affecting safety assessment, and to support a structured understanding of the link between process parameters and potential risks. The study does not aim to provide a full regulatory risk assessment, but rather to explore the suitability of this framework for the early‐stage safety evaluation of innovative bio‐based FCMs.

**FIGURE 1:**
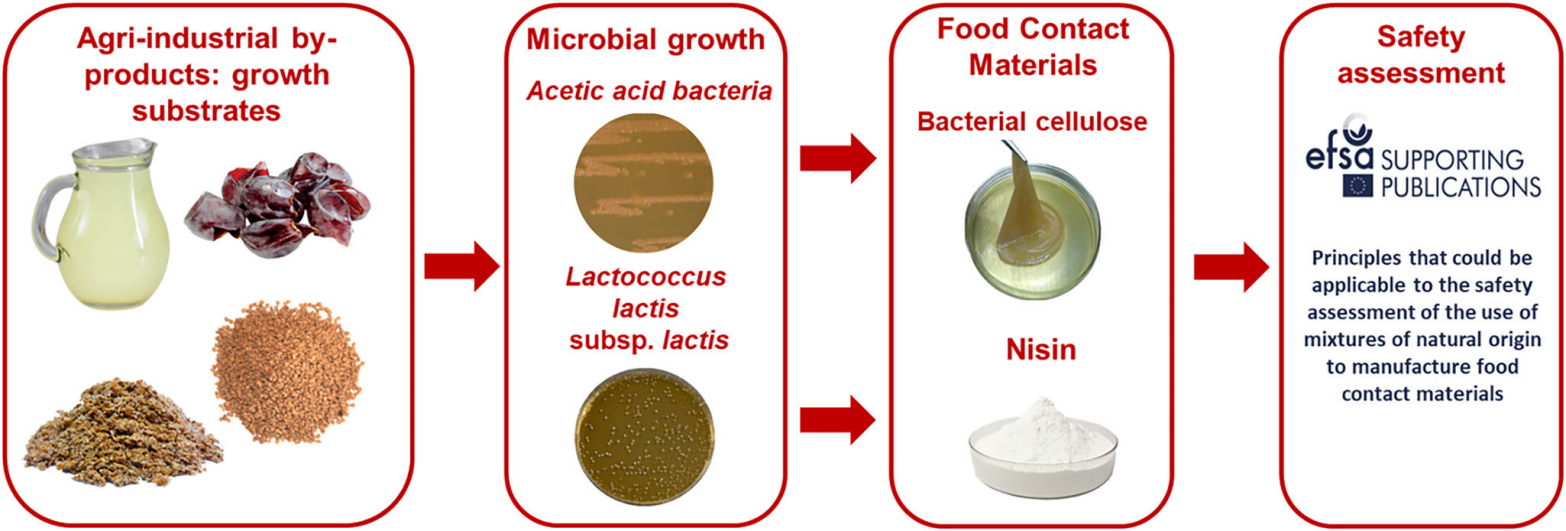
Overview of the biorefinery‐driven valorization chain converting agri‐food waste into functional biopolymers. Agro‐industrial by‐products are used as substrates for microbial fermentation, yielding bacterial cellulose (BC), and nisin, which can be incorporated into bio‐based food contact materials. The resulting food packaging solutions require a thorough safety assessment according to European Food Safety Authority (EFSA) principles.

By doing so, this study provides a practical and structured framework to support researchers, developers, and risk assessors in designing safer bio‐based FCMs and in improving their regulatory readiness (Figure 1).

Even if only the EFSA approach is described, this framework provides a comprehensive and well‐structured model for food safety evaluation within the European Union. However, many of its core principles—such as evidence‐based risk assessment, transparency, and precaution—are shared across international agencies, and the EFSA framework itself is often considered a reference model or benchmark in the development and harmonization of food safety assessment approaches worldwide.

## 2 Adopted Systematic Approach

### 2.1 Bio‐Based Food Contact Materials

Bio‐based FCMs are obtained from renewable biological resources, including plant and animal biomass, often originating as side‐streams of agri‐food production. Unlike fossil‐based plastics, these materials may provide low‐ or zero‐carbon alternatives and offer biodegradability or compostability (European Food Safety Authority (EFSA) et al. 2023). However, their natural origin does not guarantee safety, since they are often complex mixtures with variable and partly uncharacterized composition. Production may involve physical operations (extraction, purification) or microbial fermentation, both of which can intentionally or unintentionally modify their chemical profile and introduce potential safety concerns (Berenguer et al. 2023; Lee et al. 2022).

In this study, four agri‐industrial by‐products—whey, brewer's spent grain (BSG), wine marc and lees, and olive pomace—were considered as representative potential fermentation substrates. These residues are generated in large volumes by key European food chains (dairy, brewing, winemaking, and olive oil production). Their valorization supports circular economy objectives while raising relevant food safety questions due to their heterogeneous composition and possible contamination with pesticides, mycotoxins, or processing residues (García‐Bernet et al. 2024; Ladakis et al. 2020; Parfitt et al. 2018; Rao et al. 2021).

Within this context, BC and nisin were selected as case studies because they are promising bio‐based substances for food packaging applications, both obtained via microbial exploitation and already authorized or investigated for food‐related uses. Their different nature—a structural biopolymer and an antimicrobial peptide—also allows covering two complementary functional roles in FCMs: passive barrier reinforcement and active microbial inhibition (Azeredo et al. 2019; Gullo et al. 2018; Huang et al. 2024, 2025; Infante‐Neta et al. 2024; Wang et al. 2025; Xing et al. 2026). At the same time, both raise specific safety considerations linked to variability in composition, processing conditions, and potential impurities, making them suitable models for a systematic evaluation (Pandey et al. 2025; Wang et al. 2025).

BC is a natural polymer biosynthesized by *Komagataeibacter* spp. through microbial fermentation of carbohydrate substrates (Gullo et al. 2017, 2018; Njieukam et al. 2024). Compared to plant‐derived cellulose, BC is highly pure and characterized by a nanofibrillar structure with advantageous mechanical and barrier properties. Its production involves fermentation, followed by recovery and purification steps, typically including alkaline or enzymatic treatments, and possible modification processes to tailor its functionality. These stages may introduce variability in composition and potential impurities, making BC a relevant model for safety assessment in bio‐based FCMs (Sumini et al. 2025; Gullo et al. 2017).

Nisin is a ribosomally synthesized antimicrobial peptide produced by *L. lactis* subsp. *lactis*, widely used as a food preservative and increasingly considered for active packaging applications. It is produced through microbial fermentation in nutrient‐rich substrates, followed by recovery and purification steps such as precipitation or chromatographic processes. During production and processing, changes in composition, formation of by‐products, and the presence of impurities may occur, highlighting the importance of a thorough safety evaluation when used in FCMs (Bukvicki et al. 2020; Maresca and Mauriello 2022).

Overall, the use of these agri‐industrial residues as microbial substrates represents a sustainable strategy to reduce environmental impact and production costs, especially when combined with innovative recovery technologies such as enzymatic hydrolysis and membrane‐based separation (Papaioannou et al. 2022; Sharma et al. 2022; Signorello et al. 2025).

However, although bio‐based FCMs are generally associated with a lower carbon footprint compared to fossil‐derived materials, it should be noted that some processing steps required to obtain suitable material properties (e.g., chemical treatments such as alkali or acid processing) may have a significant environmental impact. Therefore, the overall sustainability of these materials should be evaluated considering the entire production process, rather than the origin of the raw materials alone.

This general description provides the technical background for the SLR carried out in this work, aimed at identifying potential substances of concern at different stages: (i) substrate composition, (ii) microbial fermentation, and (iii) downstream purification and recovery.

### 2.2 Systematic Literature Review

The SLR was performed following the main principles of the Preferred Reporting Items for Systematic Reviews and Meta‐Analyses (PRISMA) guidelines (Carrera‐Rivera et al. 2022; McCarthy et al. 2018; Page et al. 2021), in order to ensure transparency and reproducibility of the search and selection process. Research questions were formulated using relevant keywords, focusing on key process steps of interest. For the bio‐based FCMs subject of this work, the following process steps of interest were identified: natural carbon sources, microorganisms, microbial growth/fermentation, and recovery.

Research questions were identified according to the EFSA guidance document for systematic reviews concerning safety/assessments (European Food Safety Authority (EFSA) 2010) and its implementation (O'Connor et al. 2012). The approach followed the “PICO” method (acronyms of Population, Intervention, Comparator, and Outcome), which aims to develop precise and answerable research questions. The selected research questions are provided in Table 1.

**TABLE 1:** Research questions (Qs) and search structure for each of the selected process steps.

Process step of interest	Research question	Search structure
**Q_1_**. **Natural carbon source**	What are the possible toxic, hazardous constituents, contaminants, pesticide residues present in cheese whey, brewer's spent grain, wine marc, wine lees, olive pomace?	Natural products **AND** Toxic constituents
**Q_2_**. **Microorganisms**	What are the possible toxic, adverse effects of *Komagataeibacter sucrofermentans* or *Komagataeibacter xylinus/Lactococcus lactis* subsp. *lactis*?	Microorganism **AND** Adverse effects
**Q_3_**. **Microbial process**	What are the possible contaminants, impurities, residues, metabolites, and reaction/degradation products present or produced during the growth/fermentation process with *Komagataeibacter sucrofermentans* or *Komagataeibacter xylinus*/*Lactococcus lactis* subsp. *lactis* to produce bacterial cellulose/nisin?	Bacterial cellulose **AND** Production **AND** Contaminants/Nisin **AND** Production **AND** Contaminants
**Q_4_**. **Recovery**	What are the impurities, residues, and reaction products present in bacterial cellulose/nisin after purification processes? Are living cells present in the produced substance?	

Q_3_ and Q_4_ were merged due to the continuity between microbial fermentation and the subsequent recovery processes. *Komagataeibacter sucrofermentans* and *Komagataeibacter xylinus* synthesize BC as extracellular fibrils that aggregate to form a structured network (pellicle) at the air–liquid interface or within the medium, whereas *L. lactis* subsp. *lactis* produces nisin as a soluble peptide that is released directly into the culture medium.

These biopolymers are thus influenced not only by growth/fermentation conditions but also by the presence of microbial metabolites, reaction by‐products, and potential contaminants.

Following the microbial process using agri‐food by‐products such as whey, BSG, wine marc and lees, and olive pomace as substrates, the recovery and purification steps are essential to isolate BC and nisin while removing unwanted residues, processing by‐products, and, if applicable, viable microbial cells. Given that the safety of the final biopolymer is directly linked to both its production and recovery, combining Q_3_ and Q_4_ allows for a more comprehensive risk evaluation. This approach ensures that all relevant impurities, degradation products, and microbial viability concerns are assessed in a single, integrated step.

### 2.3 Literature Search Strategy and Eligibility Criteria

All the retrieved documents were obtained using a specific search string, based on the selected questions. The search string was formulated by applying the following process:

- Identification of specific keywords, considering the research question.
- Identification of synonyms.
- Use of “AND” and “OR” Boolean operators, based on predefined and selected keywords.

All the applied criteria for selecting the studies are given in Table 2. No time restrictions were applied to the literature search.

**TABLE 2:** Criteria for selecting studies related to report characteristics.

**Database (platform)**		Scopus (Elsevier; scopus.com) Web of Science (Clarivate): Science Citation Index Expanded Book Citation Index—Science Conference Abstracts Proceedings—Science Emerging Citation Index Current Chemical Resources Index Chemicus
**Language **	In	English
**Publication type **	In	Primary studies (i.e., studies generating new data). Reports from national/international risk assessment bodies and published reviews will be used to identify relevant references. Conference abstracts or posters. Reports from EU and outside EU (non‐EU) countries.
	Out	Letters to the editor Expert opinions Editorials Theses

Documents failing to meet the criteria indicated in Table 2 were excluded. The selected documents were downloaded in EndNote format and organized in a Microsoft Excel sheet format for data management; duplicate data were removed.

A refinement step was applied at this stage, consisting of the removal of duplicates, verification of record consistency across databases, and preliminary filtering based on predefined inclusion criteria (e.g., relevance to the research questions and study scope). This step was performed prior to the title and abstract screening to ensure a consistent and harmonized dataset.

**FIGURE 2:**
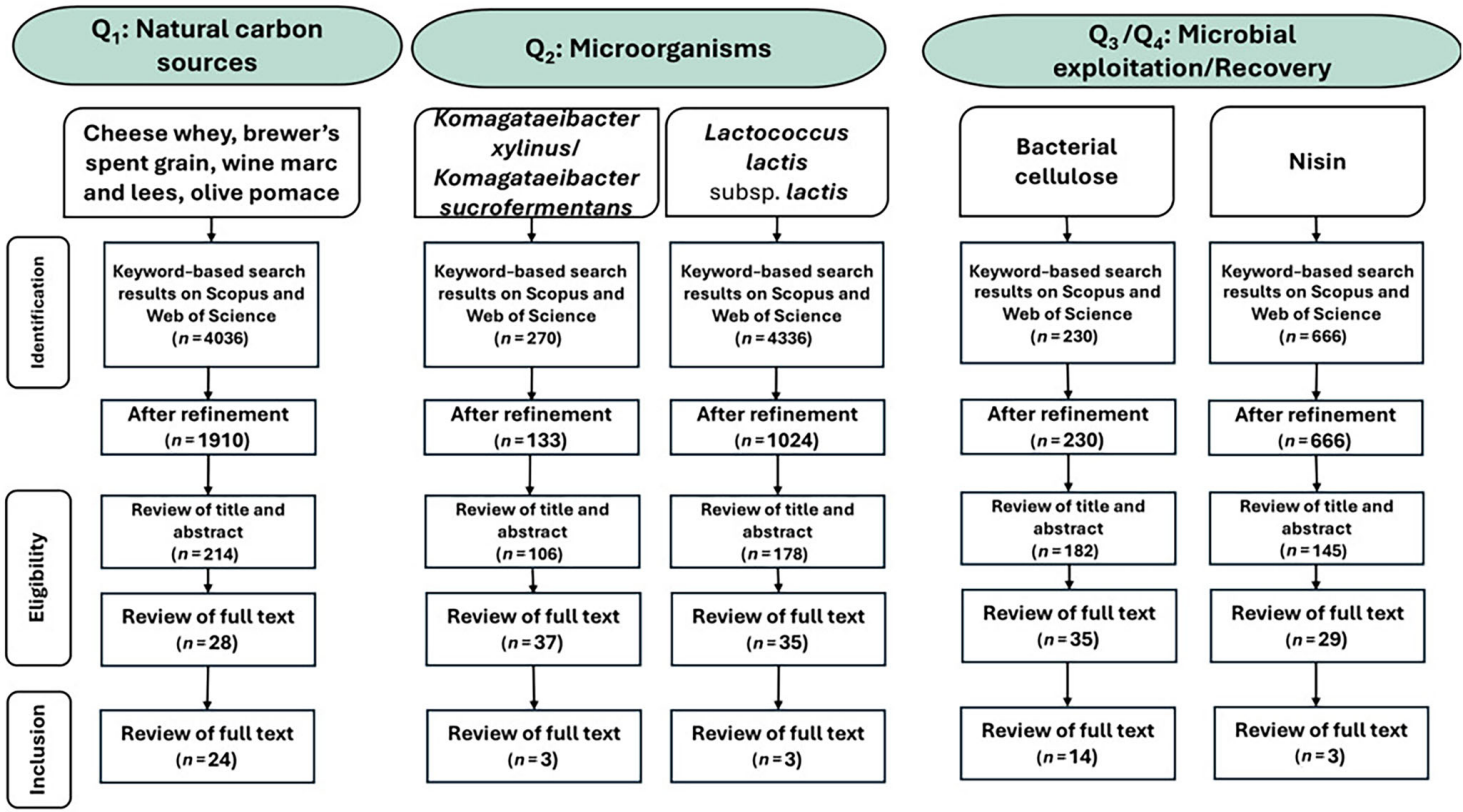
The preferred reporting items for systematic literature review, following the PRISMA methodology.

The screening of the literature took place from February 2024 until April 2024 using the predefined search strings in Scopus and Web of Science. In the retrieved scientific papers, a title and abstract (tiab) screening was performed, focusing on eligibility (relevance). Following the initial step of “tiab,” a full text search was performed to verify and conclude on article eligibility and final inclusion (Figure 2). Finally, all the included articles were screened, and data extraction was performed, focusing on the identification and presence of compounds of interest and of potential effect (toxic or allergenic) in the final bio‐based FCM. All key findings were collated and summarized to synthesize the current status of the selected compounds.

## 3 Results and Discussion

According to the EFSA Technical Report, the safety assessment of chemical mixtures of natural origin should identify potential substances of concern, starting from the natural carbon sources derived from agro‐industrial waste to the final materials and articles, at each step of the manufacturing process. The steps following the recovery process, including the use of these substances in manufacturing final bio‐based food contact articles, were beyond the scope of this work. In this study, those principles were applied to BC and nisin. A SLR was carried out to identify substances of potential concern that can potentially migrate from FCMs into food. This includes both intentionally added substances (IAS) and nonintentionally added substances (NIAS). Potential allergenicity was also included in the literature review, considering the nature of the bio‐based material and its potential effects.

A flow diagram following the PRISMA methodology, illustrating the article selection process and the number of selected articles for the SLR, is shown in Figure 2.

### 3.1 Natural Carbon Sources for Bacterial Cellulose and Nisin

A total of 1910 articles were initially identified on the selected natural carbon sources (i.e., cheese whey [CW], BSG, olive pomace, wine marc, and lees). Following the screening process, 24 articles were deemed relevant for inclusion. The subsequent sections provide a detailed analysis of each natural carbon source considered, where the selected articles are reviewed to examine the chemical composition, including the presence of key compounds and contaminants.

In addition to the presence of potential contaminants, these agri‐food by‐products can be characterized by a rich composition of fermentable carbon sources and nutrients that support microbial growth. For example, whey contains lactose and proteins, BSG provides polysaccharides and amino acids, while wine marc and olive pomace contain residual sugars, organic acids, and phenolic compounds (Balmaseda et al. 2026; Brugnoli et al. 2023a; Gottardi et al. 2023; Haring et al. 2026; Uribe‐Velázquez et al. 2026). These components can affect the biosynthesis of BC and nisin, linking the valorization of these substrates to their functional use within circular bio‐based production systems.

#### 3.1.1 Cheese Whey

Sweet whey is a by‐product of cheese production. It is composed of approximately 94% water and constitutes about 80%–90% of milk's volume, containing most water‐soluble milk components (Gottardi et al. 2023; Pereira et al. 2016; Tsermoula et al. 2021). It contains about 50% of the total milk solids, with lactose being the predominant fraction, followed by proteins, minerals, nonprotein nitrogen, and other minor compounds (Panghal et al. 2018).

Whey streams can contain chemical impurities that are introduced at different stages of the dairy supply chain, especially at the primary production level. These contaminants, including pesticides, plasticizers, and antibiotics, can enter the processing streams of whey and potentially accumulate in the final products, especially in processes such as powder production, if adequate precautions are not taken (McCarthy et al. 2018; Shelver et al. 2018). Thus, all the chemical compounds identified through the literature search, that may potentially be present in dairy products and whey are reported in Table 3.

**TABLE 3:** Chemical compounds present in dairy products and whey.

Identified risk	Substance CAS number (EC list No/ECHA)	Chemical formula	Molar mass (g/mol)	Simplified Molecular Input Line Entry System (SMILES)	Uses	Concentrations reported	References
Glyphosate (GLY)	1071‐83‐6 (EC: 213‐997‐4)	C_3_H_8_NO_5_P	169.07	C(C(=O)O)NCP(=O)(O)O	Herbicide (plant protection products)	Distribution percentage (% v/v): 0.9 ± 0.001 (in whey)	Shelver et al. (2018)
Imidacloprid (IMI)	138261‐41‐3 (EC: 428‐040‐8)	C_9_H_10_ClN_5_O_2_	255.7	C1CN(C(=N[N+](=O)[O−])N1)CC2=CN=C(C=C2)Cl	Insecticide (PPP)	Distribution percentage (% v/v): 0.9 ± 0.003 (in whey)	
Bisphenol A (BPA)	80‐05‐7 (EC:201‐245‐8)	C_15_H_16_O_2_	228.3	CC(C)(C1=CC=C(C=C1)O)C2=CC=C(C=C2)O	Plasticizer	Distribution percentage (% v/v): 0.2 ± 0.003 (in whey)	
Estrone(E1)	53‐16‐7 (EC: 200‐164‐5)	C_18_H_22_O_2_	270.4	CC12CCC3C(C1CCC2=O)CCC4=C3C=CC(=C4)O	Hormone	Distribution percentage (% v/v): 0.3 ± 0.008 (in whey)	
3′‐Methylsulfone‐2,2′,4,5,5′‐pentachloro‐biphenyl (3‐MeSO_2_‐PCB‐101)	37680‐73‐2 (EC: 621‐393‐0)	C_12_H_5_Cl_5_	326.4	C1=CC(=C(C(=C1)Cl)Cl)C2=C(C(=C(C=C2)Cl)Cl)Cl	PCB metabolite	Distribution percentage (% v/v): 0.5 ± 0.008 (in whey)	
Triclocarban (TCC)	101‐20‐2 (EC: 202‐924‐1)	C_13_H_9_Cl_3_N_2_O	315.6	C1=CC(=CC=C1NC(=O)NC2=CC(=C(C=C2)Cl)Cl)Cl	Antibacterial/disinfectant	Distribution percentage (% v/v): 0.4 ± 0.006 (in whey)	
2‐Hydroxyl‐1,3,7,8‐tetrachloro dibenzo‐*p*‐dioxin (2‐OH‐1378‐TCDD)	50585‐46‐1 (EC: ‐)	C_12_H_4_Cl_4_O_3_	338.0	C1=C2C(=CC(=C1Cl)Cl)OC3=C(C(=C(C=C3O2)Cl)O)Cl	TCDD Metabolite	Distribution percentage (% v/v): 0.6 ± 0.017 (in whey)	
2,3′,4,4′5‐Pentachlorobiphenyl (PCB‐118)	31508‐00‐6 (EC: 621‐375‐2)	C_12_H_5_Cl_5_	326.4		Coolants/plasticizers/hydraulic fluids/ pesticides/flame retardant	Distribution percentage (% v/v): 0.2 ± 0.022 (in whey)	
β‐Hexachloro‐cyclo‐dodecane (β‐HBCD)	‐ (EC: ‐)	C_12_H_18_Br_6_	641.7	C1CC(C(CCC(C(CCC(C1Br)Br)Br)Br)Br)Br	Flame retardant	Distribution percentage (% v/v): 0.2 ± 0.037 (in whey)	
1,2,7,8‐Tetrachloro dibenzo‐*p*‐dioxin(1278 – TCDD)	50585‐46‐1 (EC: ‐)	C_12_H_4_Cl_4_O_2_	322.0	C1=C2C(=CC(=C1Cl)Cl)OC3=CC(=C(C=C3O2)Cl)Cl	Industrial and incineration by‐product	Distribution percentage (% v/v): 0.3 ± 0.046 (in whey)	
Tetrabromo bisphenol A (TBBPA)	79‐94‐7 (EC: 201‐236‐9)	C_15_H_12_Br_4_O_2_	543.9	CC(C)(C1=CC(=C(C(=C1)Br)O)Br)C2=CC(=C(C(=C2)Br)O)Br	Flame retardant	Distribution percentage (% v/v): 0.6 ± 0.008 (in whey)	
2,4′,4,5,5′‐Pentabromo diphenyl ether (BDE‐99)	32534‐81‐9 (EC: ‐)	C_12_H_5_Br_5_O	564.7	C1=CC(=C(C=C1Br)Br)OC2=CC(=C(C=C2Br)Br)Br	Persistent organic pollutant	Distribution percentage (% v/v): 0.2 ± 0.045 (in whey)	
*Trihalomethanes*							Cardador et al. (2017)
Trichloromethane (TCM; chloroform)	67‐66‐3 (EC: 200‐663‐8)	CHCl_3_	119.4	C(Cl)(Cl)Cl	Detergent	n.d.–1.5 µg/L (in whey)	
Bromodichloromethane (BDCM)	75‐27‐4 (EC: 200‐856‐7)	CHBrCl_2_	163.8	C(Cl)(Cl)Br	Flame retardant	Nondetectable in whey (LOD: 0.01–0.15 µg/L)	
Dibromodichloromethane (DBCM)	594‐18‐3 (EC: 209‐829‐4)	CBr_2_Cl_2_	242.7	C(Cl)(Cl)(Br)Br	Flame retardant	n.d.–1.8 µg/L (in whey)	
						*Total THM*: n.d.‐1.8 µg/L	
*Haloacetic acids (HAA)*							
Monochloroacetic acid (MCAA)	79‐11‐8 (EC: 201‐178‐4)	C_2_H_3_ClO_2_	94.5	C(C(=O)O)Cl	Chemical intermediate	Nondetectable in whey (LOD: 0.01–0.15 µg/L)	
Dichloroacetic acid (DCAA)	79‐43‐6 (EC: 201‐207‐0)	C_2_H_2_Cl_2_O_2_	128.9	C(C(=O)O)(Cl)Cl	Chemical intermediate	n.d.–1.7 µg/L (in whey)	
Trichloroacetic acid (TCAA)	76‐03‐9 (EC: 200‐927‐2)	C_2_HCl_3_O_2_	163.4	C(=O)(C(Cl)(Cl)Cl)O	Laboratory chemical, coating product	n.d.–1.5 µg/L (in whey)	
Bromochloroacetic acid (BCAA)	5589‐96‐8 (EC: 627‐754‐9)	C_2_H_2_BrClO_2_	173.4	C(C(=O)O)(Cl)Br	Disinfectant	Nondetectable in whey (LOD: 0.01–0.15 µg/L)	
Bromodichloroacetic acid (BDCAA)	71133‐14‐7 (EC: 623‐296‐9)	C_2_HBrCl_2_O_2_	207.8	C(=O)(C(Cl)(Cl)Br)O	Disinfectant	Nondetectable in whey (LOD: 0.01–0.15 µg/L)	
						*Total HAA*: n.d.–3.2 µg/L	
N^6^‐[2‐(2‐furanyl)‐2‐oxoethyl]‐L‐Lysine (furosine)	19746‐33‐9 (EC: ‐)	C_12_H_18_N_2_O_4_	254.3	C1=COC(=C1)C(=O)CNCCCC[C@@H](C(=O)O)N	Process contaminant (Maillard reaction product)	0.84–28.8 mg/g protein (in dairy products)	Li et al. (2021)
N^ε^‐(1‐Carboxymethyl‐L‐lysine (CML)	5746‐04‐3	C_8_H_16_N_2_O_4_	204.2	C(CCNCC(=O)O)C[C@@H](C(=O)O)N	Process contaminant (AGE‐advanced glycation end‐products)	0.004–0.233 mg/g protein (20%–100% cow whey in infant formula)	
Aflatoxin M1	6795‐23‐9 (EC: 229‐865‐4)	C_17_H_12_O_7_	328.3	COC1=C2C3=C(C(=O)CC3)C(=O)OC2=C4C(=C1)O[C@@H]5[C@]4(C=CO5)O	Mycotoxin in dairy products	Milk: 0.028–0.083 µg/kg Whey: 0.017–0.055^a^	Pietri et al. (2016)
Hypochlorite	14380‐61‐1 (EC: 807‐894‐9)	ClO−	51.45	[O−]Cl	Disinfection by‐products (oxiDBP)/cleaning‐in‐process (CIP)	n.d.	McCarthy et al. (2018)
Chlorine dioxide	10049‐04‐4 (EC: 233‐162‐8)	ClO_2_	67.4	O=Cl[O]	Disinfection by‐products (oxiDBP)/cleaning‐in‐process (CIP)	n.d.	
Chlorate	14866‐68‐3 (EC:‐)	ClO_3_	83.45	[O−]Cl(=O)=O	Disinfection by‐products (oxiDBP)/cleaning‐in‐process (CIP)	n.d.	
Total mercury (Hg + methyl mercury; CH_3_Hg)	22967‐92‐6 (EC: 694‐823‐8)	Hg, CH_3_Hg	215.63	C[Hg+]	Contaminant	0.548–9.41 ng/g (whey protein)	Aquino et al. (2017)

^a^ An enrichment factor (EF) of 4.2‐5.2 during cheesemaking is reported.

The risk of contamination of dairy products and whey might also extend to cleaning‐in‐place (CIP) practices used in the maintenance of equipment in industry and agriculture. Residues of disinfectants such as quaternary ammonium compounds (QACs) and chlorine‐based disinfectants, such as hypochlorite (ClO−) and chlorine dioxide (ClO_2_), as well as biopesticides and oxidized inorganic disinfection by‐products (oxiDBPs) can remain and pose a health risk to consumers (McCarthy et al. 2018). Strict adherence to CIP protocols is critical, as whey derived from oxiDBP‐contaminated milk can contain a significant proportion (60%–85%) of the contaminants present in the original milk (Cardador et al. 2017; Li et al. 2021).

Further to the occurrence of potential contaminants, whey is also susceptible to the formation of harmful Maillard reaction products (MRPs) during thermal processing. Various compounds such as lactulosyl‐lysine (furosine), furfurals and advanced glycation end products (AGEs) can be formed during heat treatment, posing potential health risks associated with chronic diseases (Li et al. 2021). Studies have shown that harmful MRPs are present in all types of dairy products, including raw milk, albeit to varying degrees. Among processed dairy products, whey (obtained from bovine milk), cheese and condensed milk have the highest concentrations of these compounds. Interestingly, pasteurized milk and hard cheese have lower furosine levels, while whey powder and concentrated milk tend to have significantly higher concentrations. This discrepancy may be due to the higher lactose content in whey, which favors glycosylation reactions, as well as the two‐step heat treatment required for concentrated milk (pulverizing and dissolving), which may further promote the formation of MRPs (Li et al. 2021). In addition, whey can also be contaminated by various mycotoxins such as aflatoxin M_1_ (AFM_1_), which originates from lactating animals that have been fed with feed contaminated with aflatoxin B_1_ (AFB_1_). When animals eat such contaminated feed, they excrete AFM_1_ into the milk. AFM_1_, which has acute toxic effects similar to AFB_1_, has been identified as a hepatic carcinogen, albeit with lower potency compared to AFB_1_. Long‐term studies in trout and rats have revealed its carcinogenic properties, albeit with a potency of 2%–10% compared to AFB_1_. AFM_1_ has been classified by the International Agency for Research on Cancer (IARC) as a possible human carcinogen (Group 2B). The presence of AFM_1_ in dairy products, including cheese, raises significant health concerns. In cheese production, the partitioning between curds and whey usually results in AFM_1_ being retained, with about 40%–60% w/v remaining in the whey. AFM_1_ tends to bind with the protein fraction of the milk through hydrophobic interactions, resulting in higher concentrations in the cheese compared to the original milk. Studies have shown that whey contains approximately 40% less AFM_1_ than the milk used for cheese production, emphasizing the potential for AFM_1_ contamination in whey‐derived products (Pietri et al. 2016). In addition to mycotoxins, the presence of heavy metals such as total mercury (THg) in whey protein raises further concerns about product safety. In a study by Aquino et al. (2017), the THg content in 19 different brands of whey protein was analyzed. This highlighted the importance of monitoring and controlling potential contaminants throughout the dairy processing chain to protect the health and well‐being of consumers. In this study, population exposure was assessed using the hazard quotient (HQ) for mercury, which is based on the provisional tolerable weekly intake of mercury recommended by the Food and Agriculture Organization (FAO) and the World Health Organization (WHO). The HQ analysis showed that the consumption of whey protein does not pose a significant risk, as the HQ values are consistently below 1. Therefore, whey protein can be considered a healthy and safe dietary option in terms of mercury contamination (Aquino et al. 2017).

#### 3.1.2 Brewer's Spent Grain

BSG, the main by‐product of the brewing process, has a rich composition with a protein content of 15%–30% and carbohydrates up to 73%. Despite its nutritional value, BSG is often undervalued due to its high moisture content, which makes it difficult to store and transport. BSG can be used as a growth substrate for microorganisms. The latter is reported to be applied for the production of biopolymers such as BC, offering an alternative way of reusing a common by‐product of the brewing industry. However, the use of BSG is not without its challenges and considerations. The cultivation of barley and hops is often subject to attacks from bacteria, fungi, viruses, and pests that can affect the quality and yield of the crop. In particular, barley grains, which are rich in starch and storage proteins, serve as an attractive nutrient source for insects and microbial pathogens, especially during germination when amino acids, fermentable carbohydrates, and other degradation products of reserve polymers accumulate in the starchy endosperm (Pérez‐Lucas et al. 2023). Thus, all the identified chemical compounds that can potentially be present in BSG are provided in Table 4. Pests and diseases threaten cereal crops, necessitating effective weed control and pest management to ensure optimal crop health and productivity. Fungi, viruses and nematodes are considered to be the main causes of barley diseases, with fungi such as *Penicillium* and *Aspergillus* species posing additional risks during storage due to the production of mycotoxins, such as AFB_1_, deoxynivalenol (DON), zearalenone (ZEA), and ochratoxin A (OTA). Mycotoxins AFB_1_ and OTA are categorized as possibly carcinogenic to humans by the IARC (Lopes et al. 2023).

**TABLE 4:** Chemical compounds present in brewer's spent grain.

Identified risk	Substance CAS number (EC list No/ECHA)	Chemical formula	Molar mass (g/mol)	Simplified Molecular Input Line Entry System (SMILES)	Uses	Concentrations reported	References
Myclobutanil	88671‐89‐0 (EC: 410‐400‐0)	C_15_H_17_ClN_4_	288.78	CCCCC(CN1C=NC=N1)(C#N)C2=CC=C(C=C2)Cl	Pesticides (PPP)	Residual amount: 38% w/w	Pérez‐Lucas et al. (2023)
Nuarimol	63284‐71‐9 (EC: 264‐071‐1)	C_17_H_12_ClFN_2O_	314.7	C1=CC=C(C(=C1)C(C2=CC=C(C=C2)F)(C3=CN=CN=C3)O)Cl	Pesticide (PPP)	Residual amount: 42% w/w	
Propiconazole	60207‐90‐1 (EC: 262‐104‐4)	C_15_H_17_Cl_2_N_3_O_2_	342.2	CCCC1COC(O1)(CN2C=NC=N2)C3=C(C=C(C=C3)Cl)Cl	Pesticide (PPP)	Residual amount: 26% w/w	
Atrazine	1912‐24‐9 (EC: 217‐617‐8)	C_8_H_14_ClN_5_	215.68	CCNC1=NC(=NC(=N1)Cl)NC(C)C	Pesticide (PPP)	Residual amount: 55% w/w	
Terbuthylazine	5915‐41‐3 (EC: 227‐637‐9)	C_9_H_16_ClN_5_	229.71	CCNC1=NC(=NC(=N1)Cl)NC(C)(C)C	Pesticide (PPP)	Residual amount: 80% w/w	
Pendimethalin	40487‐42‐1 (EC: 254‐938‐2)	C_13_H_1_9N_3_O_4_	281.31	CCC(CC)NC1=C(C=C(C(=C1[N+](=O)[O−])C)C)[N+](=O)[O−]	Pesticide (PPP)	Residual amount: 21% w/w	
Trifluralin	1582‐09‐8 (EC: 216‐428‐8)	C_13_H_16_F_3_N_3_O_4_	335.28	CCCN(CCC)C1=C(C=C(C=C1[N+](=O)[O−])C(F)(F)F)[N+](=O)[O−]	Pesticide (PPP)	Residual amount: 17% w/w	
Fenitrothion	122‐14‐5 (EC: 204‐524‐2)	C_9_H_12_NO_5_PS	277.24	CC1=C(C=CC(=C1)OP(=S)(OC)OC)[N+](=O)[O−]	Pesticide (PPP)	Residual amount: 30% w/w	
Malathion	121‐75‐5 (EC: 204‐497‐7)	C_10_H_19_O_6_PS_2_	330.4	CCOC(=O)CC(C(=O)OCC)SP(=S)(OC)OC	Pesticide (PPP)	Residual amount: 40% w/w	
Atrazine (ATR)	1912‐24‐9 (EC: 217‐617‐8)	C_8_H_14_ClN_5_	215.68	CCNC1=NC(=NC(=N1)Cl)NC(C)C	Pesticide (PPP)	Residual amount: 60% w/w	Hack et al. (1997)
Deethylatrazine (DEA)	6190‐65‐4 (EC: 622‐793‐8)	C_6_H_10_ClN_5_	187.63	CC(C)NC1=NC(=NC(=N1)N)Cl	Pesticide (PPP)	Residual amount: 40% w/w	
Deisopropylatrazine (DIA)	1007‐28‐9 (EC: 623‐281‐7)	C_5_H_8_ClN_5_	173.60	CCNC1=NC(=NC(=N1)N)Cl	Pesticide (PPP)	Residual amount: 40% w/w	
Hydroxyatrazine (OHA)	2163‐68‐0 (EC: 690‐104‐8)	C_8_H_15_N_5_O	197.24	CCNC1=NC(=O)NC(=N1)NC(C)C	Pesticide (PPP)	Residual amount: 20% w/w	
Terbutylazine (TER)	5915‐41‐3 (EC: 227‐637‐9)	C_9_H_16_ClN_5_	229.71	CCNC1=NC(=NC(=N1)Cl)NC(C)(C)C	Pesticide (PPP)	Residual amount: 80% w/w	
Myclobutanil	88671‐89‐0 (EC: 410‐400‐0)	C_15_H_17_ClN_4_	288.78	CCCCC(CN1C=NC=N1)(C#N)C2=CC=C(C=C2)Cl	Pesticides (PPP)	1648 ± 14 µg/kg^a^ 153.83 µg^b^ (38.43% w/w)	Navarro et al. (2005)
Propiconazole	60207‐90‐1 (EC: 262‐104‐4)	C_15_H_17_Cl_2_N_3_O_2_	342.2	CCCC1COC(O1)(CN2C=NC=N2)C3=C(C=C(C=C3)Cl)Cl	Pesticide (PPP)	1772 ± 11 µg/kg^a^ 165.36 µg^b^ (41.95% w/w)	
Nuarimol	63284‐71‐9 (EC: 264‐071‐1)	C_17_H_12_ClFN_2O_	314.7	C1=CC=C(C(=C1)C(C2=CC=C(C=C2)F)(C3=CN=CN=C3)O)Cl	Pesticide (PPP)	1099 ± 3 µg/kg^a^ 102.60 µg^b^ (25.78% w/w)	
Cyproconazole	94361‐06‐5 (EC: 619‐020‐1)	C_15_H_18_ClN_3_O	291.77	CC(C1CC1)C(CN2C=NC=N2)(C3=CC=C(C=C3)Cl)O	Pesticide (PPP)	72.7 ± 8.9 µg^c^ (40.2% w/w)	Navarro et al. (2011)
Diniconazole	83657‐24‐3 (EC: 617‐485‐5)	C_15_H_17_Cl_2_N_3_O	326.2	CC(C)(C)C(C(=CC1=C(C=C(C=C1)Cl)Cl)N2C=NC=N2)O	Pesticide (PPP)	95.0 ± 7.2 µg^c^ (48.7% w/w)	
Epoxiconazol	133855‐98‐8 (EC: 106325‐08‐0)	C_17_H_13_ClFN_3_O	329.8	C1=CC=C(C(=C1)C2C(O2)(CN3C=NC=N3)C4=CC=C(C=C4)F)Cl	Pesticide (PPP)	92.2 ± 9.7 µg^c^ (43.9% w/w)	
Flutriafol	76674‐21‐0 (EC: 616‐367‐0)	C_16_H_13_F_2_N_3_O	301.29	C1=CC=C(C(=C1)C(CN2C=NC=N2)(C3=CC=C(C=C3)F)O)F	Pesticide (PPP)	63.9 ± 6.8 µg^c^ (36.4% w/w)	
Tebuconazole	107534‐96‐3 (EC: 403‐640‐2)	C_16_H_22_ClN_3_O	307.82	CC(C)(C)C(CCC1=CC=C(C=C1)Cl)(CN2C=NC=N2)O	Pesticide (PPP)	85.0 ± 6.9 µg^c^ (44.2% w/w)	
Aflatoxin B_1_	1162‐65‐8 (EC: 214‐603‐3)	C_17_H_12_O_6_	312.27	COC1=C2C3=C(C(=O)CC3)C(=O)OC2=C4C5C=COC5OC4=C1	Mycotoxin	0.4–44.5 µg/kg	Lopes et al. 2023)
Deoxynivalenol (DON)	51481‐10‐8 (EC: 610‐668‐0)	C_15_H_20_O_6_	296.31	CC1=CC2C(C(C1=O)O)(C3(CC(C(C34CO4)O2)O)C)CO	Mycotoxin	1068.0 µg/kg	
Ochratoxin A (OTA)	303‐47‐9 (EC: 206‐143‐7)	C_2_0H_18_ClNO_6_	403.8	CC1CC2=C(C=C(C(=C2C(=O)O1)O)C(=O)NC(CC3=CC=CC=C3)C(=O)O)Cl	Mycotoxin	n.d.	
Zearalenone (ZEA)	17924‐92‐4 (EC: 241‐864‐0)	C_18_H_22_O_5_	318.40	CC1CCCC(=O)CCCC=CC2=C(C(=CC(=C2)O)O)C(=O)O1	Mycotoxin	1429.0 µg/kg	
Azoxystrobin	131860‐33‐8 (EC: 603‐534‐3)	C_22_H_17_N_3_O_5_	403.41	COC=C(C1=CC=CC=C1OC2=NC=NC(=C2)OC3=CC=CC=C3C#N)C(=O)OC	Pesticide (PPP)	Rye: 15 µg/kg Barley: 11 µg/kg Wheat: 7 µg/kg	Hakme et al. (2024)
Boscalid	188425‐85‐6 (EC: 606‐143‐0)	C_18_H_12_Cl_2_N_2_O	329.80	C1=CC=C(C(=C1)C2=CC=C(C=C2)Cl)NC(=O)C3=C(N=CC=C3)Cl	Pesticide (PPP)	Barley: 47 µg/kg Wheat: 12 µg/kg	
Epoxiconazole	133855‐98‐8 (EC: 406‐850‐2)	C_17_H_13_ClFN_3_O	329.81	C1=CC=C(C(=C1)C2C(O2)(CN3C=NC=N3)C4=CC=C(C=C4)F)Cl	Pesticide (PPP)	Barley: 32 µg/kg Wheat: 7 µg/kg	
Fenpropidin	67306‐00‐7 (EC: 614‐049‐6)	C_19_H_31_N	273.5	CC(CC1=CC=C(C=C1)C(C)(C)C)CN2CCCCC2	Pesticide (PPP)	Barley: 55 µg/kg	
Fenpropimorph	67564‐91‐4 (EC: 266‐719‐9)	C_20_H_33_NO	303.5	CC1CN(CC(O1)C)CC(C)CC2=CC=C(C=C2)C(C)(C)C	Pesticide (PPP)	Rye: 194 µg/kg	
Fluquiconazole	136426‐54‐5 (EC: 411‐960‐9)	C_16_H_8_Cl_2_FN_5_O	376.2	C1=CC2=C(C=C1F)C(=O)N(C(=N2)N3C=NC=N3)C4=C(C=C(C=C4)Cl)Cl	Pesticide (PPP)	Rye: 36 µg/kg	
Flutriafol	76674‐21‐0 (EC: 616‐367‐0)	C_16_H_13_F_2_N_3_O	301.29	C1=CC=C(C(=C1)C(CN2C=NC=N2)(C3=CC=C(C=C3)F)O)F	Pesticide (PPP)	Rye: 70 µg/kg	
Fluxapyroxad	907204‐31‐3 (EC: 620‐041‐3)	C_18_H_12_F_5_N_3_O	381.3	CN1C=C(C(=N1)C(F)F)C(=O)NC2=CC=CC=C2C3=CC(=C(C(=C3)F)F)F	Pesticide (PPP)	Wheat: 8 µg/kg	
Kresoxym‐methyl	143390‐89‐0 (EC: 604‐351‐6)	C_18_H_19_NO_4_	313.3	CC1=CC=CC=C1OCC2=CC=CC=C2C(=NOC)C(=O)OC	Pesticide (PPP)	Rye: 23 µg/kg	
Spiroxamine	118134‐30‐8 (EC: 601‐505‐4)	C_18_H_35_NO_2_	297.5	CCCN(CC)CC1COC2(O1)CCC(CC2)C(C)(C)C	Pesticide (PPP)	Rye: 80 µg/kg	
Propiconazole	60207‐90‐1 (EC: 262‐104‐4)	C_15_H_17_Cl_2_N_3_O_2_	342.2	CCCC1COC(O1)(CN2C=NC=N2)C3=C(C=C(C=C3)Cl)Cl	Pesticide (PPP)	Barley: 24 µg/kg	
Prothioconazole‐desthio	120983‐64‐4 (EC: 691‐449‐7)	C_14_H_15_Cl_2_N_3_O	312.2	C1CC1(C(CC2=CC=CC=C2Cl)(CN3C=NC=N3)O)Cl	Pesticide (PPP)	Barley: 5 µg/kg	
Tebuconazole	107534‐96‐3 (EC: 403‐640‐2)	C_16_H_22_ClN_3_O	307.82	CC(C)(C)C(CCC1=CC=C(C=C1)Cl)(CN2C=NC=N2)O	Pesticide (PPP)	Barley: 25 µg/kg	
Chlorpyrifos‐methyl	5598‐13‐0 (EC: 227‐011‐5)	C_7_H_7_Cl_3_NO_3_PS	322.5	COP(=S)(OC)OC1=NC(=C(C=C1Cl)Cl)Cl	Pesticide (PPP)	Rye: 7 µg/kg	
Deltamethrin	52918‐63‐5 (EC: 258‐256‐6)	C_22_H_19_Br_2_NO_3_	505.2	CC1(C(C1C(=O)OC(C#N)C2=CC(=CC=C2)OC3=CC=CC=C3)C=C(Br)Br)C	Pesticide (PPP)	Rye: 2 µg/kg	
Mepiquat	15302‐91‐7 (EC: 604‐881‐8)	C_7_H_16_N^+^	114321	C[N+]1(CCCCC1)C	Pesticide (PPP)	Rye: 10 µg/kg	
Glyphosate	1071‐83‐6 (EC: 213‐997‐4)	C_3_H_8_NO_5_P	169.07	C(C(=O)O)NCP(=O)(O)O	Pesticide (PPP)	Rye: 170 µg/kg	
Aflatoxin B_1_	1162‐65‐8 (EC: 214‐603‐3)	C_17_H_12_O_6_	312.27	COC1=C2C3=C(C(=O)CC3)C(=O)OC2=C4C5C=COC5OC4=C1	Mycotoxin	*Medium level*: 96.6% ± 11.62 (recovery^d^ ± RSD); *High level*: 90.3 ± 10.86% (recovery ± RSD)	Benešová et al. (2012)
Aflatoxin B_2_	7220‐81‐7 (EC: 230‐618‐8)	C_17_H_14_O_6_	314.29	COC1=C2C3=C(C(=O)CC3)C(=O)OC2=C4C5CCOC5OC4=C1	Mycotoxin	*Medium level*: 93.5 ± 14.3% (Recovery ± RSD); *High level*: 93.0 ± 12.8% (recovery ± RSD)	
Aflatoxin G_1_	1402‐68‐2 (EC: 1165‐39‐5)	C_17_H_12_O_7_	328.27	COC1=C2C3=C(C(=O)OCC3)C(=O)OC2=C4C5C=COC5OC4=C1	Mycotoxin	*Medium level*: 96.1% ± 9.17 (Recovery ± RSD); *High level*: 95.5% ± 12.3 (Recovery ± RSD)	
Aflatoxin G_2_	7241‐98‐7 (EC: 230‐643‐4)	C_17_H_14_O_7_	330.29	COC1=C2C3=C(C(=O)OCC3)C(=O)OC2=C4C5CCOC5OC4=C1	Mycotoxin	*Medium level*: 101.8% ± 16.77 (Recovery ± RSD); *High level*: 74.8 ± 12.27% (Recovery ± RSD)	
Aflatoxin B_1_	1162‐65‐8 (EC: 214‐603‐3)	C_17_H_12_O_6_	312.27	COC1=C2C3=C(C(=O)CC3)C(=O)OC2=C4C5C=COC5OC4=C1	Mycotoxin	*Mean level*: i. 11.76 ± 9.15 ng/g (0 days of storage) ii. 256.96 ± 150.96 ng/g (7 days of storage)	Gerbaldo et al. (2011)
Aflatoxin B_1_	1162‐65‐8 (EC: 214‐603‐3)	C_17_H_12_O_6_	312.27	COC1=C2C3=C(C(=O)CC3)C(=O)OC2=C4C5C=COC5OC4=C1	Mycotoxin	*Average value*: *i. A. flavus*: 1.019.9 ng/g *ii. A. parasiticus*: 2.378.6 ng/g	Asurmendi et al. (2013)
Fumonisin B_1_	116355‐83‐0 (EC: 621‐436‐3)	C_34_H_59_NO_15_	721.8	CCCCC(C)C(C(CC(C)CC(CCCCC(CC(C(C)N)O)O)O)OC(=O)CC(CC(=O)O)C(=O)O)OC(=O)CC(CC(=O)O)C(=O)O	Mycotoxin	*Minimum*: 104 µg/kg *Maximum*: 145 µg/kg	Gonzalez Pereyra et al. (2011)
Aflatoxin B_1_	1162‐65‐8 (EC: 214‐603‐3)	C_17_H_12_O_6_	312.27	COC1=C2C3=C(C(=O)CC3)C(=O)OC2=C4C5C=COC5OC4=C1	Mycotoxin	*Minimum*: 19 µg/kg; *Maximum*: 44.52 µg/kg	
Fumonisin B_1_	116355‐83‐0 (EC: 621‐436‐3)	C_34_H_59_NO_15_	721.8	CCCCC(C)C(C(CC(C)CC(CCCCC(CC(C(C)N)O)O)O)OC(=O)CC(CC(=O)O)C(=O)O)OC(=O)CC(CC(=O)O)C(=O)O	Mycotoxin	*Mean level*: 226.5 µg/kg; *Minimum*: 50.30 µg/kg; *Maximum*: 908.47 µg/kg	Batatinha et al. (2007)
Deoxynivalenol (DON)	51481‐10‐8 (EC: 610‐668‐0)	C_15_H_20_O_6_	296.31	CC1=CC2C(C(C1=O)O)(C3(CC(C(C34CO4)O2)O)C)CO	Mycotoxin	*Mean*: 297.02 µg/kg; *Median*: 268.74 µg/kg;	Piacentini et al. (2019)
Zearalenone (ZEA)	17924‐92‐4 (EC: 241‐864‐0)	C_18_H_22_O_5_	318.40	CC1CCCC(=O)CCCC=CC2=C(C(=CC(=C2)O)O)C(=O)O1	Mycotoxin	*Mean*: 79.7 µg/kg; *Median*: 64.86 µg/kg	

^a^Residual levels found in the different control stages.

^b^Mean amount of pesticide in the whole weight or volume of sample for each control stage and percentage remaining.

^c^Mean amount (*n* = 3) of fungicide in the whole weight or volume of sample for each control stage and percentage remaining after mashing and boiling.

^d^Recovery was calculated using the spiked sample since the certified reference material is not commercially available for the raw materials studied.

Pesticides are widely used in crop growth and storage, but there is a shift toward integrated pest management to reduce environmental and health risks. Prudent pesticide use remains essential to maximize yields and ensure crop quality, especially in barley and hops. During the malting process, residues of pesticides applied to barley may persist and potentially transfer to downstream products such as wort and beer, albeit at varying levels depending on processing conditions. Pesticide residues present in brewing by‐products, including BSG and spent hops, might raise concerns regarding their potential impact on food safety and human health. Studies highlight the need for rigorous monitoring and mitigation of pesticide residues in brewing to ensure product safety and quality (Hakme et al. 2024; Navarro et al. 2005, 2011; Pérez‐Lucas et al. 2023).

Agreeing with Navarro et al. (2011), at the end of the mashing stage, the remaining percentages of three fungicides (myclobutanil, nuarimol, and propiconazole) were below 10% of the amount verified in malt, with propiconazole showing the greatest reduction (to 4% w/w). Contrarily, the residual amounts on spent grain were comparatively high (38%, 42%, and 26% for myclobutanil, propiconazole, and nuarimol, respectively, all the compounds having logarithm of the octanol–water partition coefficient [log KOW] > 2). Comparable behavior was noted for atrazine and terbuthylazine during mashing, when 55% w/w and 80% w/w, respectively, were retained on spent grains (Hack et al. 1997). As a general rule, adsorption affinity is directly related to their polarities: the more polar the pesticide, the lower the adsorbed amount observed. It is important to point out that malt and adjuncts maceration generates a high amount of suspended matter, which could adsorb residues and, if the detected levels allow it, the spent grains are commonly used as animal feeds, which implies a commercial practice for this by‐product. The residual amounts of dinitroaniline herbicides nearly disappear after mashing (< 1% of the initial amount in sweet wort), while the remaining percentages of organophosphorus insecticides, fenitrothion and malathion, in sweet wort were greater, at 4% and 7%, respectively (Navarro et al. 2005). Contrarily, the recovered amounts on spent grain were quite high (21%, 17%, 30%, and 40% for pendimethalin, trifluralin, fenitrothion, and malathion, respectively). Other water‐soluble pesticides, such as glyphosate (organophosphorus) and pirimicarb (carbamate), were found in sweet wort in amounts higher than 80%, while no residues of pyrethroid compounds (fenvalerate, deltamethrin, permethrin, and flucythrinate) were detected in sweet wort because they were retained on the spent grains. For oxamyl, dichlorvos, parathion‐methyl, chlorpyrifos, dichlofuanid, and captafol, noticeable losses were observed, possibly due to evaporation, thermal degradation, and/or chemical reactions with some wort components during mashing (Navarro et al. 2005). Azole fungicides (cyproconazole, diniconazole, epoxiconazole, flutriafol, myclobutanil, propiconazole, tebuconazole, and triadimenol) were recovered from the sweet wort in proportions varying from 3% to 36% for diniconazole and triadimenol, respectively, while about 40%–50% of the initial mass on malt was found in spent grains (Navarro et al. 2005).

Studies to assess mycotoxin contamination in BSG and brewer's spent yeast (BSY) from EU breweries found traces of aflatoxins (AFs), with only one sample of BSG and BSY testing positive for AF contamination, in particular AFB_1_, at concentrations of 0.4 µg/kg and 0.2 µg/kg, respectively (Benešová et al. 2012). Conversely, BSG samples of Argentinian origin showed higher levels of AFB_1_ contamination, ranging from 11.76 µg/kg to 257.0 µg/kg, with prevalence rates of up to 57% (Gerbaldo et al. 2011). In addition, surveys revealed varying levels of AF contamination in BSG destined for pig feed, with concentrations of up to 50.4 µg/kg and prevalence rates of 31.3% (Asurmendi et al. 2013). In addition, studies have detected fumonisins (FBs) in BSG samples, with concentrations ranging from 19 µg/kg to 145 µg/kg, emphasizing the importance of comprehensive monitoring of mycotoxins in brewing by‐products (Gonzalez Pereyra et al. 2011). For FBs, a survey assessing their presence in BSG from Brazil, intended for incorporation into dairy cattle feed, reported concerning findings. Approximately 72.5% of all samples (total of 80) were found to be contaminated with FBs, with an average concentration of 226.5 µg/kg and the minimum‐maximum concentrations to be 50.30 µg/kg and 908.47 µg/kg, respectively (Batatinha et al. 2007). This finding underscores the importance of monitoring mycotoxin contamination in brewing by‐products to prevent potential health risks to livestock consuming contaminated feed.

Furthermore, investigations into the presence of DON and ZEA in Brazilian BSG and BSY revealed notable contamination levels. BSG samples exhibited an average concentration of 1068.0 µg/kg for DON and 1429.0 µg/kg for ZEA, highlighting the significance of assessing mycotoxin levels in brewing by‐products for human and animal health. These findings emphasize the need for stringent quality control measures to mitigate mycotoxin contamination risks associated with brewing by‐products (Lopes et al. 2023; Piacentini et al. 2019).

Additionally, an investigation of contamination with OTA in Brazilian malt BSG reported no detectable contamination in the samples studied (Pires et al. 2019). While this is reassuring, it again highlights the importance of continued monitoring and control measures to ensure the safety and quality of brewing by‐products utilized in various applications, including potentially the use for manufacturing FCMs.

In the context of sustainable beer production, a comprehensive assessment of potential hazards such as pesticide residues is essential. Hakme et al. (2024) investigated the fate of pesticides during the brewing process and showed that, on average, 58% w/w of pesticide residues are found in brewing by‐products, with the highest fraction (53% w/w) found in the spent grain (Hakme et al. 2024). in particular, nonpolar pesticides tended to remain adsorbed on the spent grain during the brewing process, while polar pesticides such as glyphosate and mepiquat showed different behavior, with the majority (> 80% w/w) migrating into the beer. A significant proportion of pesticide residues that were originally introduced to the brewery from rye were found in the spent grain. For fenpropimorph and spiroxamine, a recovery percentage greater than 90% w/w was observed in the spent grain. The concentration of chlorpyrifos‐methyl, kresoxym‐methyl, and fluquinconazole in the spent grain was observed to be between 60% w/w and 70% w/w of their original concentration. This suggests that a significant amount of these pesticide residues was retained in the spent grain during the brewing process. A fraction of less than 50% w/w was observed for azoxystrobin, deltamethrin, and flutriafol in spent grain. The concentration of glyphosate and mepiquat was even lower, with only 17% w/w and 31% w/w, respectively, retained in the spent grain. When considering the initial concentrations of pesticide residues found in barley, it was observed that 78% w/w of propiconazole and 75% w/w of prothioconazole‐desthio ended up in the spent grain. A ratio between 60% w/w and 70% w/w was observed for boscalid, epoxiconazole, propiconazole, and tebuconazole in the spent grain. A lower fraction of 55% w/w was observed in spent grain for azoxystrobin (Hakme et al. 2024). These results underline the importance of monitoring pesticide residues in both final products and by‐products to ensure consumer safety and the environmental sustainability of beer production, or the sustainable use of these by‐products for manufacturing FCMs.

In conclusion, evaluating the composition and contamination levels of BSG is crucial for its safe and sustainable use in various applications.

#### 3.1.3 Wine Marc and Lees

In the context of exploring natural carbon sources for the production of biopolymers through microbial fermentation, wine marc and lees emerge as potential substrates. However, their application requires careful consideration due to the presence of contaminants and toxins that may affect the safety of resulting biopolymer‐based FCMs.

Winery by‐products, including grape pomaces, have come under scrutiny due to the presence of mycotoxins, particularly OTA. A study conducted in the Douro Demarcated Region, Portugal, by Ribeiro and Alves (2008) implemented an analytical methodology for OTA quantification in grape pomaces. The method involved solvent extraction followed by high‐performance liquid chromatography (HPLC) with fluorescence detection, revealing OTA concentrations not exceeding 0.4 µg kg−1 in samples from 12 out of 13 sites. Although the detected levels were below regulated thresholds, the study underscores the necessity of establishing regulatory frameworks for winery by‐products, especially those intended for alternative uses like animal feed or biopolymer production.

The vinification process introduces the potential for pesticide residues in grape‐derived products. Monitoring during the 2007 vintage, as described by Čuš et al. (2010), revealed residues of various pesticides in grapes, must, lees, and wine. Persistent pesticides such as boscalid and phosalone were detected, albeit significantly diminished through the processing stages. The concentrations of pesticides detected in the wine marc and lees is 0.09 ± 0.04 mg/kg for boscalid in white grapes and 0.06 ± 0.01 mg/kg in red grapes, 0.43 ± 0.03 mg/kg in white grapes for cyprodinil, 0.21 ± 0.01 mg/kg in white grapes and 0.04 ± 0.00 mg/kg for red grapes for dimethomorph, 0.63 ± 0.03 mg/kg in white grapes for phosalone, 0.01 ± 0.00 mg/kg in red grapes for fenhexamid, 1.09 ± 0.05 mg/kg for procymidone, 0.90 ± 0.03 mg/kg for pyrimethanil in white grapes, 0.04 ± 0.00 mg/kg for tubonazole in white grapes. These findings emphasize the importance of thorough assessment and mitigation strategies during vinification to ensure the safety of the final products. Among the pesticides detected in the lees, fenhexamid and procymidone were prominently present. Fenhexamid was found at concentrations exceeding 0.20 mg per kg of lees, indicating its significant accumulation in this vinification by‐product. Procymidone was also detected at relatively high concentrations in the lees, highlighting its affinity toward this substrate. Additionally, other pesticides were detected in the lees, including boscalid, cyprodinil, dimethomorph, phosalone, and pyrimethanil. These pesticides exhibited varying concentrations in the lees, with some exceeding regulatory limits for safety in food products. The study of Čuš et al. (2010) highlighted the efficacy of certain vinification processes, such as pressing and separation of lees from wine after alcoholic fermentation, in significantly reducing pesticide residues. This observation underscores the importance of these processing steps in mitigating the potential transfer of pesticides from grape‐derived products to the final wine. Furthermore, the analysis of Pinot Gris grapes revealed specific pesticide residues detected in the wine after racking and storage, with boscalid, dimethomorph, and procymidone being prominent. The concentrations of these residues in the final wine were found to be significant, albeit within regulatory limits (Čuš et al. 2010).

Concluding for wine marc and less, regulatory standards, analytical methodologies, and risk mitigation strategies established for winery by‐products can inform safety assessments for biopolymer‐based FCMs derived from such substrates. Among the identified chemical components potentially present in this particular waste stream, also environmental contaminants such as trace metals and organic pollutants pose concerns (e.g., originating from soil, water, or agricultural inputs), cannot be excluded. Moreover, potential contamination of wine marc and lees by mycotoxins (Ribeiro and Alves 2008), pesticide residues, and other undesirable chemicals cannot be excluded, as already reported (see Table 5). Their suitability as growth substrates for microorganisms, especially for microbial biopolymer production, must be carefully assessed and evaluated.

**TABLE 5:** Chemical compounds present in wine marc and lees.

Identified risk	Substance CAS number (EC list No/ECHA)	Chemical formula	Molar mass (g/mol)	Simplified Molecular Input Line Entry System (SMILES)	Uses	Concentrations reported	References
Ochratoxin A (OTA)	303‐47‐9 (EC: 206‐143‐7)	C_20_H_18_ClNO_6_	403.8	CC1CC2=C(C=C(C(=C2C(=O)O1)O)C(=O)NC(CC3=CC=CC=C3)C(=O)O)Cl	Mycotoxin	Maximum: 0.1 µg/kg Average: 0.07 µg/kg	Ribeiro and Alives (2008)
Boscalid	188425‐85‐6 (EC: 606‐143‐0)	C_18_H_12_Cl_2_N_2_O	343.2	C1=CC=C(C(=C1)C2=CC=C(C=C2)Cl)NC(=O)C3=C(N=CC=C3)Cl	Pesticide (PPP)	White grapes: 0.09 ± 0.04 mg/kg Red grapes: 0.06 ± 0.01 mg/kg	Čuš et al. (2010)
Cyprodinil	121552‐61‐2 (EC: 601‐785‐8)	C_14_H_15_N_3_	225.29	CC1=CC(=NC(=N1)NC2=CC=CC=C2)C3CC3	Pesticide (PPP)	White grapes: 0.43 ± 0.03 mg/kg	
Dimethomorph	110488‐70‐5 (EC: 404‐200‐2)	C_21_H_22_ClNO_4_	387.9	COC1=C(C=C(C=C1)C(=CC(=O)N2CCOCC2)C3=CC=C(C=C3)Cl)OC	Pesticide (PPP)	White grapes: 0.21 ± 0.01 mg/kg Red grapes: 0.04 ± 0.00 mg/kg	
Phosalone	2310‐17‐0 (EC: 218‐996‐2)	C_12_H_15_ClNO_4_PS_2_	367.8	CCOP(=S)(OCC)SCN1C2=C(C=C(C=C2)Cl)OC1=O	Pesticide (PPP)	White grapes: 0.63 ± 0.03 mg/kg	
Procymidone	32809‐16‐8 (EC: 251‐233‐1)	C_13_H_11_Cl_2_N_2_	284.13	CC12CC1(C(=O)N(C2=O)C3=CC(=CC(=C3)Cl)Cl)C	Pesticide (PPP)	White grapes: 1.09 ± 0.05 mg/kg	
Pyrimethanil	53112‐28‐0 (EC: 414‐220‐3)	C_12_H_13_N_3_	199.25	CC1=CC(=NC(=N1)NC2=CC=CC=C2)C	Pesticide (PPP)	White grapes: 0.90 ± 0.03 mg/kg	
Tebuconazole	107534‐96‐3 (EC: 403‐640‐2)	C_16_H_22_ClN_3_O	307.82	CC(C)(C)C(CCC1=CC=C(C=C1)Cl)(CN2C=NC=N2)O	Pesticide (PPP)	White grapes: 0.04 ± 0.00 mg/kg	
Fenhexamid	126833‐17‐8 (EC: 422‐530‐5)	C_14_H_17_Cl_2_NO_2_	302.20	CC1(CCCCC1)C(=O)NC2=C(C(=C(C=C2)O)Cl)Cl	Pesticide (PPP)	Red grapes: 0.01 ± 0.00 mg/kg	

#### 3.1.4 Olive Pomace

In the realm of microbial fermentation for biopolymer production utilizing agro‐industrial by‐products, olive pomace emerges as a promising substrate for the growth of the microorganisms. Olive pomace is a by‐product resulting from olive oil extraction. Throughout history, agricultural practices, including olive cultivation, have grappled with challenges posed by pests and weeds, leading to the application of various pesticides for crop protection. However, the indiscriminate use of pesticides has sparked concerns regarding their residues in food products, particularly in essential commodities like olive oil. The presence of pesticides or residues or contaminants in olive oil, or in olive pomace, can potentially pose health risks to consumers (Schmidt et al. 2023; Waqas et al. 2021).

Furthermore, olive products are vulnerable to contamination by mycotoxins, which are secondary metabolites produced by filamentous fungi. Mycotoxins, such as AFs and ZEA, have been found to contaminate olive pomace oil (Abdolmaleki et al. 2021). Hence, contamination of olive pomace with mycotoxins, might of importance regarding food safety and necessitating thorough monitoring and control measures.

In the assessment of contaminants in olive products, polycyclic aromatic hydrocarbons (PAHs) represent another significant class of concern reported in the literature. Guillén et al. (2004) conducted a study to investigate the occurrence of PAHs in olive pomace oil, aiming to determine the contamination degree and evaluate if any specific purification steps are necessary during its manufacture. The study applied gas chromatography–mass spectrometry (GC–MS) for the analysis and identified a very high number (71) of PAHs, with a wide range of molecular weights and aromatic ring numbers in five samples (OP_1_, OP_2_, OP_3_, OP_4_, OP_5_), highlighting the risk of certain PAHs being present at low concentration (280.35–831.75 µg/kg) levels in olive pomace (Guillén et al. 2004). Moreda et al. (2004) developed a method for the determination of high molecular mass PAHs in refined olive pomace and other vegetable oils. Their method involved solid‐phase extraction (SPE) followed by high‐performance liquid chromatography with fluorescence detection (HPLC‐FLD). The study reported satisfactory recoveries (> 80%) and detection limits ranging from 0.01 to 0.2 µg/kg for various PAHs in different vegetable oils, including olive pomace oil (Moreda et al. 2004; see Table 6).

**TABLE 6:** Chemical and biological compounds present in olive pomace (OP).

Identified risk	Substance CAS number (EC list No/ECHA)	Chemical formula	Molar mass (g/mol)	Simplified Molecular Input Line Entry System (SMILES)	Uses	Concentrations reported	References
Aflatoxin B_1_	1162‐65‐8 (EC: 214‐603‐3)	C_17_H_12_O_6_	312.27	COC1=C2C3=C(C(=O)CC3)C(=O)OC2=C4C5C=COC5OC4=C1	Mycotoxin	20 samples of olive oil: 8.51 µg/kg	Schmidt et al. (2023) Waqas et al. (2021) Lin et al. (2022)
Alternariol	641‐38‐3 (EC: 636‐339‐1)	C_14_H_10_O_5_	258.23	CC1=CC(=CC2=C1C3=C(C(=CC(=C3)O)O)C(=O)O2)O	Mycotoxin	12.5–13.7 µg/kg	
Zearalenone (ZEA)	17924‐92‐4 (EC: 241‐864‐0)	C_18_H_22_O_5_	318.40	CC1CCCC(=O)CCCC=CC2=C(C(=CC(=C2)O)O)C(=O)O1	Mycotoxin	OP oil; Mean level: 0.7 µg/kg *Crude olive oil*; *Mean level*: 0.6 µg/kg	Abdolmaleki et al. (2021)
Aflatoxin G_2_	7241‐98‐7 (EC: 230‐643‐4)	C_17_H_14_O_7_	330.29	COC1=C2C3=C(C(=O)OCC3)C(=O)OC2=C4C5CCOC5OC4=C1	Mycotoxin	*Crude olive oil*: 1.4‐6.8 µg/kg	
80 different PAHs (see footnote^c^)					PAHs	280.35 µg/kg ‐ 831.75 µg/kg	Guillén et al. (2004)
Benz[a]anthracene (BaA)	56‐55‐3 (EC: 200‐280‐6)	C_18_H_12_	228.3	C1=CC=C2C(=C1)C=CC3=CC4=CC=CC=C4C=C32	PAHs	Refined OP A: < LOQ^a^ Refined OP B: 0.14 µg/kg Refined OP C: 0.11 µg/kg	Moreda et al. (2004)
Chrysene (CHR)	218‐01‐9 (EC: 205‐923‐4)	C_15_H_26_N_8_O_4_S	414.5	C1=C(NC=N1)CC(C(=O)NC(CCCN=C(N)N)C(=O)O)NC(=O)C(CS)N	PAHs	Refined OP A: 0.14 µg/kg Refined OP B: 0.26 µg/kg Refined OP C: 0.43 µg/kg	
Benzo(e)pyrene (BeP)	91259‐16‐4 (EC: ‐)	C_20_H_11_NO_2_	297.3	C1=CC=C2C(=C1)C3=CC=CC4=C3C5=C(C=C4)C=CC(=C25)[N+](=O)[O−]	PAHs	Refined OP A: < LOQ^a^ Refined OP B: 0.80 µg/kg Refined olive pomace C: 1.98 µg/kg	
Benzo(b)fluoranthene (BbFA)	205‐99‐2 (EC: 110‐497‐7)	C_20_H_12_	252.3	C1=CC=C2C3=C4C(=CC=C3)C5=CC=CC=C5C4=CC2=C1	PAHs	Refined OP A: < LOQ^a^ Refined OP B: < LOQ^a^ Refined OP C: < LOQ^a^	
Benzo(k)fluoranthene (BkFA)	207‐08‐9 (EC: 205‐916‐6)	C_20_H_12_	252.3	C1=CC=C2C=C3C4=CC=CC5=C4C(=CC=C5)C3=CC2=C1	PAHs	Refined OP A: < LOQ^a^ Refined OP B: < LOQ^a^ Refined OP C: 0.15 µg/kg	
Benzo(a)pyrene (BaP)	91259‐16‐4 (EC: ‐)	C_20_H_12_	297.3	C1=CC=C2C3=C4C(=CC2=C1)C=CC5=C4C(=CC=C5)C=C3	PAHs	Refined OP A: ND^b^ Refined OP B: < LOQ^a^ Refined OP C: 0.34 µg/kg	
DBahA	53‐70‐3 (EC: 200‐181‐8)	C_22_H_14_	278.3	C1=CC=C2C(=C1)C=CC3=CC4=C(C=CC5=CC=CC=C54)C=C32	PAHs	Refined OP A: ND^b^ Refined OP B: < LOQ^a^ Refined OP C: < LOQ^a^	
BghiP	191‐24‐2 (EC: 205‐883‐8)	C_22_H_12_	276.3	C1=CC2=C3C(=C1)C4=CC=CC5=C4C6=C(C=C5)C=CC(=C36)C=C2	PAHs	Refined OP A: ND^b^ Refined OP B: 0.40 µg/kg Refined OP C: 0.62 µg/kg	
IP	193‐39‐5(EC: 205‐893‐2)	C_22_H_12_	276.3	C1=CC=C2C(=C1)C3=C4C2=CC5=CC=CC6=C5C4=C(C=C6)C=C3	PAHs	Refined OP A: ND^b^ Refined OP B: ND^b^ Refined OP C: < LOQ^a^	

Abbreviation: PAHs, polycyclic aromatic hydrocarbons.

^a^ LOQ, limit of quantification; ^b^ND, not detected; ^c^Naphthalene, acenaphthene, acenaphthylene, fluorene, phenanthrene, anthracene, fluoranthene, pyrene, benzo[c]phenanthrene, benz[a]anthracene, chrysene, 7, 12‐dimethylbenz[a]anthracene, benzo[b]fluoranthene, benzo[j]fluoranthene, benzo[k]fluoranthene, benzo[a]pyrene, 3‐methylcholanthrene, indeno[1, 2, 3‐cd]pyrene, dibenz[a, h]anthracene, benzo[ghi]perylene, dibenzo[a, l]pyrene, dibenzo[a, i]pyrene, and dibenzo[a, h]pyrene, 7‐dimethylnaphthalene, 1, 4‐dimethylnaphthalene, 1, 5‐dimethylnaphthalene, 1‐methylphenanthrene, 3, 6‐dimethylphenanthrene, 2, 3‐dimethylanthracene, 9, 10‐dimethylphenanthrene, 2‐methylfluoranthene, 1‐methylfluoranthene, 11H‐benzo[c]fluorene, 1‐methylpyrene, 6‐methylbenz[a]anthracene, 7‐methylbenz[a]anthracene, 3‐methylchrysene, 2‐methylchrysene, 5‐methylchrysene, 4‐methylchrysene, 6‐methylchrysene, 1‐methylchrysene, dibenz[a, j]anthracene, benzo[b]chrysene, picene, anthanthrene, coronene, and dibenzo[a, e]pyrene, 1, 6‐dimethylnaphthalene, 2, 6‐dimethylnaphthalene, o‐terphenyl, 2‐methylanthracene, 9‐methylanthracene, m‐terphenyl, 11*H*‐benzo[a]fluorene, 11*H*‐benzo[b]fluorene, benzo[e]‐pyrene, and perylene.

In summary, the utilization of olive pomace as a substrate for microbial exploitation to produce biopolymers underscores the necessity for comprehensive safety assessments to address potential contaminant residues, including pesticides, mycotoxins, and PAHs. Understanding the interplay between olive oil polyphenolic compounds and contaminants is crucial for ensuring the safety and quality of olive‐derived products. Future research efforts should concentrate on developing effective strategies for contaminant mitigation and elucidating the intricate interactions between polyphenolic compounds and contaminants in olive products.

Research on by‐products involves the development of sensitive methodologies to detect contaminants at low levels and the creation of efficient procedures to minimize hazardous substances in the final products. Understanding how processing steps impact the presence or degradation of new contaminants is also crucial. Similarly, challenges arise during the extraction of biopolymers from these by‐products, as the extraction processes may inadvertently coextract contaminants or other compounds alongside substances of interest. Therefore, it is essential to evaluate the presence, bioactivity, and occurrence of hazardous substances to ensure the safety of the final biopolymer products. Overall, a comprehensive understanding of contaminant risks in food by‐products, such as olive pomace, among others, is essential for developing safe and sustainable valorization strategies without compromising human health or the environment. However, technological limitations arise, including the diverse compositions of by‐products complicating the purification and recovery of polymers, and challenges in maintaining sustainability due to the variability in agricultural waste production throughout the year.

### 3.2 Microorganisms for the Production of Bacterial Cellulose and Nisin

#### 3.2.1 Microorganisms for the Production of Bacterial Cellulose

A total of 106 publications were reviewed to assess the safety of *K. sucrofermentans* and *K. xylinus*. This process led to the final selection of two articles, one EFSA Scientific Opinion, and one EFSA Statement for *K. sucrofermentans*, and one article for *K. xylinus*.

##### 3.2.1.1 Komagataeibacter sucrofermentans

*K. sucrofermentans*, formerly referred to as *Acetobacter xylinus* subsp. *sucrofermentans* and later as *Gluconacetobacter sucrofermentans*, has undergone several taxonomic reclassifications. Cleenwerck et al. (2010) analyzed strains within the genus *Gluconacetobacter*, including *Gluconacetobacter xylinus* LMG 18788, previously regarded as the type strain of *Acetobacter xylinus* subsp. *sucrofermentans*. Their study demonstrated that this strain represents a distinct species, thus supporting its reclassification as *Gluconacetobacter sucrofermentans*. This clarification is relevant because it ensures that safety assessments are applied to the correct taxonomic entity and that data reported under previous names can still be validly considered while avoiding misinterpretation.

Safety assessments of *K. sucrofermentans* have been conducted with a focus on its potential implications for food and feed safety. Notably, EFSA has evaluated the safety of this microorganism as part of its QPS process (EFSA Panel on Biological Hazards (BIOHAZ) et al. 2019, 2020). During this approach, experts assess the taxonomic identity of the microorganism, the related body of knowledge and potential safety concerns. The assessment revealed no reported safety issues associated with the use of *K. sucrofermentans*, and no antimicrobial resistance aspects have been documented for this species. Consequently, *K. sucrofermentans* (formerly *A. xylinus* subsp. *sucrofermentans*) was proposed for the QPS list with the qualification “QPS applies for production purposes only.” According to the EFSA Technical Report on natural compounds, this indicates that there is no safety concern for the microorganism itself, unless the biomass or carbon source used introduces potentially toxic contaminants.

##### 3.2.1.2 Komagataeibacter xylinus

*K. xylinus* has also been investigated with regard to its potential applications, particularly in biotechnology and health sciences. In a study by Lavasani et al. (2019), the safety and probiotic properties of a particular strain K.X.1 were evaluated for possible use in the management of obesity and diabetes. The strain showed a high glucose conversion rate and significant survival in acidic pH and bile salt environments, indicating its suitability as a probiotic. Safety assessments in rats included daily monitoring of general health, food and water intake, body weight (bw), and the occurrence of diarrhea or vomiting, as well as histopathological examination of liver, intestine, and spleen tissues. Bacterial translocation from the gut to distant organs was also assessed. No adverse clinical signs, differences in feed or water consumption, weight changes, bacterial translocation, inflammation, degeneration, or necrosis were observed in the treated rats compared to controls.

Based on the reviewed literature, no major toxic or adverse effects were reported for *K. sucrofermentans* and *K. xylinus* in the contexts studied. For *K. sucrofermentans*, EFSA evaluations indicate no safety concerns under the QPS qualification “for production purposes only,” while for *K. xylinus*, animal studies showed no adverse clinical or histopathological effects. These findings support their potential use in BC production, but caution is warranted due to the limited number of studies and the variability in strains and production conditions. Further research would be valuable to comprehensively assess potential safety concerns, particularly for human applications and under diverse fermentation conditions.

#### 3.2.2 Microorganisms for the Production of Nisin

A total of 1024 articles were analyzed to assess the safety of *L. lactis* subsp. *lactis*, leading to the selection of two EFSA Scientific Opinions, which consider three articles to evaluate the safety concerns.

##### 3.2.2.1 *Lactococcus lactis* Subsp. *lactis*

EFSA conducted two scientific opinions to assess the safety concerns associated with *L. lactis* (EFSA Panel on Biological Hazards (BIOHAZ) et al. 2017, 2019). In the EFSA Panel on Biological Hazards (EFSA Panel on Biological Hazards (BIOHAZ) et al. 2019) 173 references were considered for the QPS assessment, from which 6 articles were selected for further analysis based on their potential to raise safety concerns. After reviewing the abstracts, four papers were identified that might raise safety concerns. However, further analysis revealed that one paper (Kato et al. 2018) did not address any safety issues, while the remaining three did (Chen et al. 2018; Kaboré et al. 2018; Shimizu et al. 2019).

In two of the selected papers (Chen et al. 2018; Kaboré et al. 2018), phenotypical methods were used to identify the etiological agent, which are known to be unreliable for this species. Moreover, the other two studies (Chen et al. 2018; Shimizu et al. 2019) described cases in which clear predisposing conditions were identified. For example, Shimizu et al. (2019) reported a case of *L. lactis* cholangitis in a patient with cholangiocarcinoma, which obstructed bile evacuation, and a bilioduodenal catheter was inserted. This predisposing condition may have been the source of the infection. Similarly, Kaboré et al. (2018) described the microbiota of 125 cases of endodontitis, where *L. lactis* was present as part of a polymicrobial community in five cases, suggesting oral colonization rather than being the causative agent of infection.

The overall assessment concluded that there are no significant safety concerns regarding the use of *L. lacti*s in food and feed applications, provided it adheres to the conditions outlined in the QPS framework. EFSA supports its continued use with no additional risk to consumer health. While rare instances of *L. lactis* presence have been reported in clinical settings, these cases are typically linked to predisposing health conditions, and under normal circumstances, the microorganism poses a low risk of adverse effects. Consequently, the QPS recommendation for *L. lactis* remains valid, based on the limited evidence of infections and the absence of broader safety concerns in the literature.

### 3.3 Biotechnological Process and Recovery Steps

#### 3.3.1 Bacterial Cellulose

To assess the microbial and recovery processes for BC production, a total of 230 scientific articles were screened, which resulted in the final selection of 14 articles.

##### 3.3.1.1 Biotechnological Process

In specific culture conditions (temperature, pH, and nutrient availability), BC is produced at the cytoplasmic membrane and exuded as nanofibrils, which aggregate into microfibrils and further assemble into ribbon‐shaped fibrils (Cielecka et al. 2019). Numerous studies have focused on using agro‐industrial wastes as alternative culture media, which not only reduce production costs but also align with circular economy principles (Azeredo et al. 2019).

For food‐related applications, BC has been studied as thickening and gelling agent, stabilizer, water‐binding additive and also as a packaging material. Its production typically involves fermentation followed by recovery of the pellicle and purification steps (Shahmohammadi Jebel and Almasi 2016). The safety of BC for potential use in food applications and as FCMs in food packaging and coatings formulation was evaluated in several studies. BC is currently produced and marketed on a large scale and has a long history of use in Asian countries under the trade name “Nata de coco,” and was exported to Europe, the United States, and the Middle East between 2006 and 2013. It can be produced from a variety of *Komagataeibacter* strains and has generally recognized as safe (GRAS) status in the United States since 1992 (Dourado et al. 2017). BC was demonstrated GRAS in the United States as a food ingredient through the independent conclusion process under 21 Code of Federal Regulations (CFR) 182.1 using scientific procedures in accordance with 201 (s) (21 USC Section 321 (s)) of the Federal Food, Drug and Cosmetic Act. A GRAS affirmation petition was filed on the basis of the GRAS determination of BC on December 11, 1991, which was accepted by the Food and Drug Administration (FDA) in 1992 (U.S. Food and Drug Administration 1992). GRAS status in the United States is granted based on a self‐determination process, where scientific data and evidence are provided to support that a substance is safe for its intended use in food. The FDA reviews the information and, if no concerns arise, the substance is recognized as safe for use in food. In contrast, the inclusion of *K. sucrofermentans* in the EFSA's QPS list reflects a different regulatory approach. QPS status is granted by EFSA for microorganisms intentionally added to food, based on a comprehensive assessment of available scientific data that suggest no safety concerns under specific conditions. The QPS list applies to the microorganism itself, not to the substance produced by it. Furthermore, an application for the authorization of a BC aqueous suspension (*HydroFibre*) was submitted to the Food Safety Authority of Ireland (FSAI) by Satisfibre S.A. of Portugal in accordance with Article 4 of the novel food Regulation (EC) No 258/97. This application was accepted by the FSAI on December 19th of 2016.

*HydroFibre*, produced with *K. sucrofermentans*, is chemically identical to plant cellulose and consists of ribbon‐shaped microfibrils in a hydrated paste (5% cellulose, 95% water), free from lignin and pectin. It is intended for use in various foods (e.g., composite foods, meat, dairy, juices, ice cream, bakery products) at 2%–20% inclusion levels. Safety assessments relied on data for microcrystalline cellulose (MCC) due to compositional similarity. Estimated intakes of BC from *HydroFibre* are 397 mg/kg bw/day on average and 902 mg/kg bw/day in high intake scenarios, well below the established ADI of 7 g/kg bw/day. The margins of safety are 16‐fold for toddlers and eightfold for combined exposure with MCC (E460) in children.

In 2017, a review of the toxicological and dietetic aspects of BC in animal models concluded that BC from *Komagataeibacter* is safe for food applications (Dourado et al. 2017). Studies on acute and subacute oral toxicity in various animal models further support this conclusion. Available toxicological data, including in vitro tests, indicate that BC is not genotoxic, carcinogenic, tumor‐promoting, pyrogenic, or a developmental or reproductive toxicant (Jagannath et al. 2010; Pinto et al. 2016). In addition, the data available support the general conclusion that BC is nontoxic on ingestion, skin contact or on inhalation, or elicit any other inflammatory or oxidative stress responses at the cellular level (Napavichayanun et al. 2018).

A comprehensive review by Girard et al. (2024) highlights potential impurities in nanocellulose (NC)‐based biomaterials and emphasizes their impact on biosafety in medical and pharmaceutical applications. Two main sources of impurities are identified: endogenous, from the cellulosic raw materials and bacterial synthesis (proteins, metabolic by‐products such as acetic acid, lipopolysaccharides, and residual compounds from the fermentation medium), and exogenous, introduced during processing and handling (microbial contaminants, endotoxins, chemical residues, or additives like pH regulators and plant debris). While high purity is critical for medical applications to avoid immune responses or inflammation, food, agricultural, or textile applications can tolerate lower levels. The purification and recovery process of BC—typically involving physical extraction, washing, and chemical or enzymatic treatments—aims to remove or reduce both endogenous and exogenous impurities, although residual traces may remain depending on the method used.

Liu et al. (2018) studied the levels of potentially immunogenic contaminants, such as endotoxin and (1,3)‐β‐D‐glucan, in four representative types of NCs, including wood‐based NCs and BC. The high contamination levels of these contaminants in BC are primarily attributed to insufficient production control, particularly in the process of nata de coco. Compared to wood‐derived NCs, BC exhibits nearly 3 times higher (1,3)‐β‐D‐glucan contamination, which leachable endotoxin levels in unpurified BC (Nata de coco) reaching up to 450 EU/g BC (Liu et al. 2018). Additionally, heavy metals such as lead (Pb), cadmium (Cd), mercury (Hg), arsenic (As), and copper (Cu) have been detected within the BC matrix, likely originating from environmental contamination or from natural carbon sources used as fermentation substrates. Although BC is generally regarded as biocompatible and nontoxic, its source, preparation methods, and chemical modifications significantly influence its final properties and toxicological profile. To ensure safety in food, biomedical, and pharmaceutical applications, strict aseptic measures must be implemented throughout production, storage, and processing to minimize contamination risks (Li et al. 2024).

Pigaleva et al. (2019) illustrated the development of a novel method for simultaneous purification and sterilization of BC films through a high‐pressure process, including supercritical conditions with carbon dioxide (CO_2_). In the process, the BC films (produced by *G. hansenii*) were exposed to supercritical CO_2_ in the presence of Milli‐Q water, promoting the dissolution of CO_2_ and the formation of carbonic acid. For comparison, the authors also employed three conventional methods for bacterial cell removal: 5% solutions of sodium dodecyl sulfate (SDS), sodium bicarbonate (NaHCO_3_), and radioimmunoprecipitation assay (RIPA) buffer. BC films were immersed in these solutions for 3 days, with the solutions replaced every 24 h. After treatment, the films were washed thoroughly with distilled water (at least 20 times over 5 days) until the pH of the washing solution reached neutral (pH 7.0), after which they were stored in water at 5°C. To assess the effectiveness of all these treatments, the processed BC samples were subjected to various analytical techniques, including atomic force microscopy (AFM), scanning electron microscopy (SEM), energy‐dispersive X‐ray spectroscopy (EDS), and infrared (IR) spectrometry. The results revealed that the conventional methods (5% solutions of SDS, NaHCO_3_, and RIPA buffer) often fail to completely remove cells and endotoxins, leaving traces of washing compounds in the samples. Additionally, the high‐pressure CO_2_ processing successfully destroyed the outer lipopolysaccharide membranes of the bacterial cells, but the cells still retained their shape and dimensions. Although the high‐pressure CO_2_ method reduced endotoxin levels, the remaining endotoxins were still above the FDA‐recommended safety threshold of 0.025 EU/mg, making BC unsuitable for medical applications (Pigaleva et al. 2019).

Furthermore, in another study, the efficacy of different alkaline rinsing methods in removing bacteria and bacterial metabolites from cellulose matrices produced by three strains of *K. xylinus* was evaluated. SEM analysis also revealed that rinsing with sodium hydroxide (NaOH) did not result in the complete removal of *K. xylinus* cells. Although the microbial cells did not survive this strong alkaline treatment, some bacterial structures remained intact in the cellulose matrix. These residual structures could trigger immune responses, inflammation, or cytotoxicity, as supported by experiments showing effects on fibroblasts, osteoblasts, and macrophage oxidative stress. Higher NaOH concentrations increased macrophage activation and reactive oxygen species (ROS) production. The study highlights that even if the bacteria are nonviable, their components can affect biocompatibility. An optimized rinsing protocol is therefore needed to minimize bacterial residues while preserving cellulose properties, with important implications for biomedical applications such as wound dressings. The study did not report on the removal of heavy metals, which remains a consideration for material safety (Junka et al. 2017).

Gromovykh et al. (2019) evaluated three washing methods for removing bacterial cells: 5% of SDS, RIPA, and RIPA with nucleases. AFM confirmed the removal of bacterial cells by all methods. Quantitative analysis revealed a significant reduction in deoxyribonucleic acid (DNA) content with RIPA buffer and nucleases compared to the nonwashed matrix. Live/Dead assay demonstrated BC's adhesive nature and ability to maintain cell viability. Overall, washing with 5% SDS and RIPA is effective, but the addition of nucleases is required to minimize residual bacterial DNA (Gromovykh et al. 2019; see Table 7).

**TABLE 7:** Substances of concern in bacterial cellulose (BC).

Identified risk	Substance CAS Number (EC list No/ECHA)	Chemical formula	Molar mass (g/mol)	Simplified Molecular Input Line Entry System (SMILES)	Production technology	Uses	Concentrations reported	References
Lipid A (endotoxin)	67924‐63‐4 (EC: 881‐019‐9)	C_211_H_376_N_8_O_126_P_6_	5227	CCCCCCCCCCCCCC(=O)OC(CCCCCCCCCCC)CC(=O)OC1C(C(OC(C1OP(=O)(O)O)COC2(CC(C(C(O2)C(CO)O)OC3C(C(C(C(O3)C(CO)O)OP(=O)(O)OP(=O)(O)OCCN)OC4C(C(C(C(O4)C(COC5C(C(C(C(O5)C(CO)O)OP(=O)(O)O)O)O)O)O)OC6C(C(C(C(O6)COC7C(C(C(C(O7)CO)O)O)O)O)OC8C(C(C(C(O8)CO)O)O)OC9C(C(C(C(O9)CO)O)OC1C(C(C(C(O1)COC1C(C(C(C(O1)CO)O)O)NC(=O)C)O)OC1C(C(C(C(O1)CO)O)OC1C(C(C(C(O1)COC1C(C(C(C(O1)CO)O)O)O)O)O)O)NC(=O)C)NC(=O)C)OC1C(C(C(C(O1)CO)O)O)NC(=O)C)O)O)O)OC1(CC(C(C(O1)C(CO)O)O)OC1(CC(C(C(O1)C(CO)O)O)OP(=O)(O)OCCN)C(=O)O)C(=O)O)C(=O)O)OCC1C(C(C(C(O1)OP(=O)(O)O)NC(=O)CC(CCCCCCCCCCC)O)OC(=O)CC(CCCCCCCCCCC)O)O)NC(=O)CC(CCCCCCCCCCC)OC(=O)CCCCCCCCCCC		Endotoxin	450 EU/g (Li et al. 2024; Liu et al. 2018) 27 EU/mL (Girard et al. 2024; Pigaleva et al. 2019) < 0.25 EU/mL (Dourado et al. 2017)	Li et al. (2024) Liu et al. (2018) Girard et al. (2024) Pigaleva et al. (2019) Dourado et al. (2017)
(1,3)‐β‐D‐glucan	9041‐22‐9 (EC: 906 + 632‐8)	C_18_H_32_O_16_	504.4	C(C1C(C(C(C(O1)OC2C(OC(C(C2O)O)OC3C(OC(C(C3O)O)O)CO)CO)O)O)O)O		Glucan	29.73 µg/g µg/g (Liu et al. 2018)	
Living cells of acetic acid bacteria and residual bacterial DNA	n.a.						Endotoxin level before processing in Untreated (control): 2.14 EU/mg Endotoxin level after processing in scCO_2_: 1.36 EU/mg Endotoxin level after processing in Carbonic acid (biphase system): > 0.68 EU/mg AFM images confirm that the lipopolysaccharide membranes are not completely destroyed and washed out. (Pigaleva et al. 2019) Residual bacterial DNA: Untreated = 12.2 ± 3.2 ng RIPA = 9.7 ± 6.5 ng RIPA + nucleases = 2.5 ± 0.98 ng (Gromovykh et al. 2019)	Pigaleva et al. (2019) Junka et al. (2017) Gromovykh et al. (2019)

In response to legislation, scientific developments, and public concerns, toxicity‐testing methods have been implemented to generate information on the potential hazards or risks posed to humans by the use of several agents. Due to primary concerns on the toxicity, cancer and reproductive development of human food substances, their placement on the market is often dependent on Regulatory approval.

#### 3.3.2 Nisin

Nisin is highly effective against several pathogenic Gram‐positive bacteria, including *Staphylococcus aureus* (Wang et al. 2020), *Listeria monocytogenes* (Zhao et al. 2020), and *Clostridium tyrobutyricum* (Ávila et al. 2020). It also demonstrates activity against some Gram‐negative pathogens such as *Salmonella enterica*, and *Pseudomonas fluorescens* (Liang et al. 2020; Novickij et al. 2020) in combination with other hurdles. Pure nisin or nisin preparations can be obtained by culturing nisin‐producing strains of *L. lactis* subsp. *lactis*, followed by suitable extraction and purification methods. Advances in purification processes have led to the identification of several types of nisin have been identified (A, Z, and Q), each exhibiting distinct biological activities (Khelissa et al. 2021).

Moreover, it is important to highlight that nisin, when produced ex situ, cannot be regarded as a “natural” preservative when used in concentrations higher than those naturally found in foods fermented with nisin‐producing strains. When used for food preservation purposes, nisin can be directly added to food products (Aasen et al. 2000) or incorporated in packaging films (Dıblan and Kaya 2018) in different amount and products in relation to country existing regulations. The effect of direct addition of free nisin in dairy products is widely studied from a research point of view. Pinto et al. (2011) added nisin during Serro cheese manufacturing process against *Staphylococcus aureus* contamination. Pinto et al. (2011) observed that nisin did not affect the physicochemical and mechanical characteristics of the obtained cheese. In the same way, Yoon and Kim (2022) studied the inactivation of *Listeria monocytogenes* after adding nisin to the whole, low fat and skim milk. Their results showed that the anti‐*Listeria* activity of nisin was dependent on fat contents in milk substrate. The anti‐*Listeria* activity was moderate in whole milk, whereas remarkable in low‐fat and skim milk samples. The reaction between nisin and the listerial cell membrane was caused by hydrophobic interaction between amino acid residues of nisin and the fatty acids of the membrane phospholipids. Also, several researchers combined nisin with other antimicrobial agents such as essential oil (Siroli et al. 2016), chitosan films (Cé et al. 2012) or with other antimicrobial treatments such as high‐pressure processing (Marcos Muntal et al. 2013). The second strategy for nisin use as a food preservative is its incorporation into polymeric films. The advantage of using bacteriocins in films, instead of their direct addition into food matrices, is associated with the increased stability of nisin and the control of its release (Barbosa et al. 2013). Nevertheless, as free nisin application, the effectiveness of antimicrobial packaging is dependent on the type of food packed, the film‐forming polymer, and the type and concentration of antimicrobial that will determine the release rate and therefore, the antimicrobial efficiency (Marcos Muntal et al. 2013). For example, Barbosa et al. (2013) formulated cellulose films containing nisin to improve the safety of minimally processed mangoes. Many studies have demonstrated the antimicrobial potential of polymeric gels, films, or plastic polymers containing nisin, but very few studies have reported the antimicrobial potential of immobilized living cells, potentially bacteriocin producers, on selected pathogenic bacteria (Brachkova et al. 2010; Concha‐Meyer et al. 2011; Gialamas et al. 2010; Millette et al. 2004).

Nisin is a food additive (preservative), thus does not require toxicological data for its assessment to be used to manufacture plastic FCMs, but only data on its identity, uses and potential migration. However, it was decided to keep the search and related questions 3 and 4 to better understand the data available on potential substances of concern coming from or eliminated from the microbial fermentation and the recovery.

Out of 666 publications reviewed, only three met the selection criteria, including two EFSA opinions and one study addressing the economic and environmental aspects of nisin production from agri‐food by‐products. Nisin is produced through microbial fermentation followed by recovery and purification steps, which may involve the use of chemical agents (e.g., hydrochloric acid [HCl] or ammonium sulfate), potentially leading to residual substances of concern.

Traditionally, nisin is produced through microbial fermentation in the late exponential growth phase of *L. lactis*, commonly used as a starter culture in dairy products. However, the current production process is associated with high costs compared to chemical‐based alternatives, limiting its market competitiveness. The study of Arias et al. (2021) explores the economic feasibility and environmental impacts of biotechnological coproduction of nisin and lactic acid using three food‐associated industrial agro‐food by‐products: CW, sugar beet pulp (SBP), and corn stover (CS).

During the production of nisin from by‐products of the agri‐food industry, several chemical compounds are used that may be of concern and that may later be present in the final product, such as HCl for the acidification step or ammonium sulfate ((NH_4_)_2_SO_4_) for the precipitation step. Furthermore, in the context of developing sustainable packaging, the incorporation of nisin into BC using cocultivation techniques is considered by Huang et al. (2024). Through cocultivation of *K. xylinus* and *L. lactis* subsp. *lactis*, BC/nisin films with enhanced antibacterial activity and mechanical properties are successfully produced. Cocultivation is a fermentation strategy employed in biotechnology to reduce costs, allowing the simultaneous cultivation of microorganisms, such as BC‐producing strains and those contributing functional components. This approach aims to produce functional cellulose efficiently. Microbial cocultivation can boost yields and create new compounds but requires different growth conditions and media.

The EFSA evaluated nisin's safety and antimicrobial resistance implications in October 2006 (European Food Safety Authority (EFSA) 2006). Nisin, a food preservative authorized by Directive 95/2/EC, is used in products like ripened and processed cheese, puddings, clotted cream, and mascarpone. Despite concerns about antimicrobial resistance, the EFSA panel did not find any evidence to warrant changing the acceptable daily intake (ADI) of 0.13 mg/kg bw, as previously established. The panel highlighted that nisin's longstanding use and its inactivation by digestive enzymes ensure its safety. The potential for resistance to therapeutic antibiotics from nisin‐resistant mutants was deemed low due to distinct differences in their antimicrobial mechanisms. Later in October 2006, the EFSA's Scientific Panel on Food Additives reviewed nisin's safety in a new food category, liquid eggs, and evaluated a modified production process (European Food Safety Authority (EFSA) 2006). The modified process involved fermenting a sugar medium instead of milk, resulting in purer nisin with fewer residues. The panel confirmed that this new production method did not alter the previously established ADI (0.13 mg/kg bw). Additionally, this method mitigated risks for individuals with milk allergies. The panel found no safety concerns regarding the use of nisin in liquid eggs at a concentration of up to 6.25 mg/L. In October 2017, EFSA reassessed nisin's safety in light of new toxicological data and proposed its use in un‐ripened cheese and heat‐treated meat products (EFSA Panel on Food Additives and Nutrient Sources Added to Food (ANS) et al. 2017). Previously evaluated in 2006, nisin's ADI was 0.13 mg/kg bw. However, a new toxicity study, in which rats were administered nisin A for 90 days, identified a no observed adverse effect level (NOAEL) of 225 mg/kg bw/day. Based on this study, EFSA recalculated an ADI of 1 mg/kg bw/day, applying a default uncertainty factor of 200. EFSA's exposure estimates, derived from the EFSA Comprehensive Database, showed that overall exposure to nisin remained below the new ADI for all population groups. Consequently, the panel concluded that extending nisin's use to unripened cheese (up to 12 mg/kg) and heat‐treated meat products (up to 25 mg/kg) posed no safety concerns. EFSA's comprehensive evaluations over the years have consistently affirmed the safety of nisin (E234) as a food additive, even with new data and expanded uses. The adjustments in ADI and considerations for new food categories and production methods reflect ongoing vigilance in ensuring public health safety. Moreover, being authorized as a food additive, the safety assessment of nisin for use in manufacturing plastic FCMs only requires data on its identity, intended use, migration, migration of its impurities, breakdown, and reaction products; no toxicological data are required (European Food Safety Authority (EFSA) et al. 2023).

### 3.4 Allergenicity

Bio‐based polymers, derived from renewable biological sources such as plants, animals, and microorganisms, are increasingly used due to their sustainability and reduced environmental impact compared to petroleum‐based polymers. However, potential allergenicity must be considered in their development and application.

Pure cellulose is generally considered nonallergenic due to its inert nature and absence of proteins, which are the primary cause of allergic reactions. Nevertheless, allergenic risks may arise from impurities or additives introduced during production. In the context of this study, BC is produced from agri‐food by‐products such as whey, BSG, wine marc and lees, and olive pomace. These substrates may contain allergenic proteins (e.g., dairy proteins, yeast‐derived compounds, or plant allergens), which could persist as residues if not adequately removed during fermentation and purification processes (Mossburger and Scherf 2024; Romão et al. 2022).

Evidence from the literature also highlights that cellulose derivatives may act as hidden allergens in specific cases. For example, Townsend et al. (2021) reported a rare case of anaphylaxis associated with carboxymethyl cellulose (CMC, E466), used as a food and pharmaceutical additive. This case illustrates the importance of considering not only intrinsic allergenicity but also the role of processing‐derived modifications and impurities, as well as the challenges associated with ingredient identification due to variable nomenclature.

Nisin is GRAS by regulatory authorities, with limited evidence of allergenicity (EFSA Panel on Food Additives and Nutrient Sources Added to Food (ANS) et al. 2017; European Food Safety Authority (EFSA) 2006). However, potential immune responses or cross‐reactivity with other proteins cannot be entirely excluded, although such effects are not well documented (Musiejuk and Kafarski 2023).

In addition to intrinsic and process‐related factors, allergen migration from bio‐based FCMs represents an emerging concern. Recent studies have demonstrated that FCMs derived from allergenic raw materials (e.g., wheat or rye) can release allergens such as gluten into food under specific conditions, with migration levels influenced by factors such as contact time, temperature, and pH (Mossburger and Scherf 2024). Although the materials considered in this study differ from cereal‐based FCMs, similar concerns may arise if allergenic substrates are used during fermentation.

In particular, agri‐food by‐products such as CW may introduce allergens like casein and β‐lactoglobulin (Monaci et al. 2024; Xie et al. 2024), while cereal‐derived residues may contain gluten. If not adequately controlled, these components may persist in the final material and potentially migrate into food.

Overall, the allergenic risk associated with bio‐based FCMs is primarily linked to residual contaminants from substrates and processing steps rather than to the materials themselves. Therefore, appropriate control strategies should be implemented throughout the production chain, including careful selection and characterization of substrates, optimization of fermentation conditions, and effective purification processes to minimize residual allergens. In addition, clear labeling and consideration of regulatory gaps related to allergen declaration for FCMs are important to ensure consumer protection, particularly for sensitive populations.

## 4 Conclusion

This SLR examined the potential risks associated with the use of bio‐based FCMs derived from microbiological processes aimed at the valorization of agricultural wastes, focusing on two representative case studies: BC and nisin. The importance of a rigorous safety assessment for these innovative materials is emphasized, particularly in the context of the circular economy and the increasing demand for sustainable packaging solutions.

BC, produced by *Komagataeibacter* spp., offers significant advantages due to its renewable and biodegradable nature. However, this study highlights the need to carefully characterize the natural carbon sources used as substrates. The QPS status of the microorganisms should also be considered. In addition, metabolites and fermentation by‐products must be evaluated. Potential impurities, including contaminants originating from agricultural practices, should be assessed. Finally, the migration of these substances into food needs to be investigated. A full safety assessment covering all these aspects was beyond the scope of this study.

Regarding nisin, an antimicrobial additive produced by *L. lactis* subsp. *lactis*, this review highlights different but equally important challenges. Particular attention should be given to the production process, possible chemical changes during processing, and the presence of impurities and reaction products. In addition, migration behavior and interactions with packaging matrices should be considered. Compared to BC, the assessment of nisin benefits from existing regulatory evaluations as a food additive. However, its application in FCMs still requires specific considerations.

Overall, the findings emphasize the importance of adopting a comprehensive and structured approach to the safety assessment of bio‐based FCMs derived from agro‐industrial by‐products. The EFSA Technical Report provides a valuable framework for the evaluation of materials derived from mixtures of natural origin. A key aspect is that substances of natural origin cannot be assumed to be safe a priori. Their compositional complexity requires detailed characterization of all components that may migrate into food.

A major outcome of this study is the recognition of the relevance of NIAS. These substances may originate from substrates, microbial metabolism, or processing steps. Their identification remains challenging due to the complexity and variability of bio‐based systems. From a regulatory perspective, NIAS represent a significant gap, requiring advanced analytical approaches and case‐by‐case assessment. Addressing this issue is essential for ensuring a comprehensive safety evaluation of bio‐based FCMs.

## 5 Future Perspective

Future research should address aspects that were beyond the scope of the present study, particularly the implementation of full safety assessments covering all stages of the production chain.

In particular, further work is needed to improve the identification and characterization of NIAS, including the development of advanced analytical methods for the detection and quantification of impurities and degradation products. Strategies for monitoring these substances throughout the material lifecycle should also be developed.

Moreover, interdisciplinary collaboration among experts in chemistry, toxicology, microbiology, and materials science will be essential to support a comprehensive and integrated safety evaluation. Increasing awareness among producers and consumers regarding potential risks and best practices for the safe use of bio‐based FCMs will also play a key role in facilitating their safe and effective implementation.

## Nomenclature

ADI: acceptable daily intake
AFs: aflatoxins
AFB_1_
: aflatoxin B1
AFM: atomic force microscopy
AFM_1_
: aflatoxin M1
AGEs: advanced glycation end products
ANS: EFSA Panel on Food Additives and Nutrient Sources added to Food
BC: bacterial cellulose
BIOHAZ: EFSA Panel on Biological Hazards
BSG: brewer's spent grain
BSY: brewer's spent yeast
bw: body weight
CFR: Code of Federal Regulations
CIP: cleaning‐in‐place
CMC: carboxymethyl cellulose
COVID‐19: Coronavirus disease 2019
CS: corn stover
CW: cheese whey
DHA: dehydroalanine
DHB: dehydrobutyrine
DNA: deoxyribonucleic acid
DON: deoxynivalenol
EC: European Community
EDS: energy‐dispersive X‐ray spectroscopy
EFSA: European Food Safety Authority
EU: European Union
EU (endotoxin units): endotoxin units
FAO: Food and Agriculture Organization of the United Nations
FCMs: food contact materials
FDA: (US) Food and Drug Administration
FSAI: Food Safety Authority of Ireland
GC–MS: gas chromatography–mass spectrometry
GRAS: generally recognized as safe
HPLC: high‐performance liquid chromatography
HPLC–FLD: high‐performance liquid chromatography with fluorescence detection
HQ: hazard quotient
IARC: International Agency for Research on Cancer
IAS: intentionally added substances
IR: infrared (spectrometry)
MCC: microcrystalline cellulose
MRPs: Maillard reaction products
NCs: nanocelluloses
NIAS: nonintentionally added substances
NOAEL: no observed adverse effect level
OTA: ochratoxin A
PAHs: polycyclic aromatic hydrocarbons
PICO: population, intervention, comparator, and outcome
PRISMA: Preferred Reporting Items for Systematic Reviews and Meta‐Analyses
QACs: quaternary ammonium compounds
QPS: qualified presumption of safety
RIPA: radioimmunoprecipitation assay
ROS: reactive oxygen species
SBP: sugar beet pulp
scCO_2_
: supercritical carbon dioxide
SDS: sodium dodecyl sulfate
SEM: scanning electron microscopy
SLR: systematic literature review
SPE: solid‐phase extraction
THg: total mercury
tiab: title and abstract
WHO: World Health Organization
ZEA: zearalenone

## Author Contributions

**Marianna Ciccone**: conceptualization, writing – original draft, investigation, methodology, formal analysis, writing – review and editing, visualization. **Emmanouil Tsochatzis**: writing – original draft, supervision, data curation, validation, software, writing – review and editing, conceptualization, investigation, methodology. **Joel Armando Njieukam**: investigation, formal analysis, methodology. **Lorenzo Siroli**: writing – review and editing, supervision, data curation, conceptualization, investigation, validation. **Davide Gottardi**: conceptualization, investigation, writing – review and editing, validation, data curation, supervision. **Rosalba Lanciotti**: supervision, writing – review and editing, conceptualization, validation, investigation. **Francesca Patrignani**: writing – review and editing, project administration, resources, supervision, conceptualization, validation, funding acquisition, investigation.

## Funding

This research was supported by the Giordana Masetti award, established by Europass—Regional Liaison Structure with EFSA—in partnership with EFSA and the University of Parma, to enable doctoral students from universities in the Emilia‐Romagna Region to carry out research training at EFSA.

## Disclosure

The views expressed in this publication are those of the authors and should not be interpreted as representing the official position of the European Food Safety Authority (EFSA). The present article is published under the sole responsibility of the authors and may not be considered as an EFSA scientific output. EFSA cannot be held accountable for any errors or inaccuracies that may appear.

## Conflicts of Interest

The authors declare no conflicts of interest.

## References

[crf370480-bib-0001] Aasen, I. M. , T. Møretrø , T. Katla , L. Axelsson , and I. Storrø . 2000. “Influence of Complex Nutrients, Temperature and pH on Bacteriocin Production by *Lactobacillus sakei* CCUG 42687.” Applied Microbiology and Biotechnology 53, no. 2: 159–166. 10709977 10.1007/s002530050003

[crf370480-bib-0002] Abdolmaleki, K. , S. Khedri , L. Alizadeh , F. Javanmardi , C. A. F. Oliveira , and A. M. Khaneghah . 2021. “The Mycotoxins in Edible Oils: An Overview of Prevalence, Concentration, Toxicity, Detection and Decontamination Techniques.” Trends in Food Science & Technology 115: 500–511. 10.1016/j.tifs.2021.06.057.

[crf370480-bib-0003] Adeyeye, S. A. O. , and P. Sankarganesh . 2026. “Applications of Biopolymers as Sustainable Materials in Value‐Added and Functional Food Packaging: A Review.” Nutrition & Food Science 56, no. 1: 114–142. 10.1108/NFS-03-2025-0126.

[crf370480-bib-0004] Aquino, L. F. M. C. D. , R. D. O. R. Ribeiro , J. S. Simoes , S. B. Mano , E. T. Mársico , and C. A. Conte Junior . 2017. “Mercury Content in Whey Protein and Potential Risk for human Health.” Journal of Food Composition and Analysis 59: 141–144. 10.1016/j.jfca.2017.02.014.

[crf370480-bib-0005] Arias, A. , G. Feijoo , and M. T. Moreira . 2021. “Process and Environmental Simulation in the Validation of the Biotechnological Production of Nisin From Waste.” Biochemical Engineering Journal 174: 108105. 10.1016/j.bej.2021.108105.

[crf370480-bib-0006] Asurmendi, P. , C. Barberis , A. Dalcero , L. Pascual , and L. Barberis . 2013. “Survey of *Aspergillus* Section *Flavi* and Aflatoxin B1 in Brewer's Grain Used as Pig Feedstuff in Córdoba, Argentina.” Mycotoxin Research 29, no. 1: 3–7. 10.1007/s12550-012-0148-5. 23334719

[crf370480-bib-0007] Ávila, M. , N. Gómez‐Torres , P. Gaya , and S. Garde . 2020. “Effect of a Nisin‐Producing Lactococcal Starter on the Late Blowing Defect of Cheese Caused by *Clostridium tyrobutyricum* .” International Journal of Food Science & Technology 55, no. 10: 3343–3349. 10.1111/ijfs.14598.

[crf370480-bib-0008] Azeredo, H. M. C. , H. Barud , C. S. Farinas , V. M. Vasconcellos , and A. M. Claro . 2019. “Bacterial Cellulose as a Raw Material for Food and Food Packaging Applications.” Frontiers in Sustainable Food Systems 3: 7. 10.3389/fsufs.2019.00007.

[crf370480-bib-0009] Balmaseda, A. , N. Rozès , M. T. Lisanti , C. Reguant , and C. Nioi . 2026. “From Waste to Worth: Wine Lees Composition and Applications in Research and Industry.” Critical Reviews in Food Science and Nutrition 66, no. 2: 369–391. 10.1080/10408398.2025.2525441. 40601530

[crf370480-bib-0010] Barbi, S. , M. Brugnoli , S. La China , M. Montorsi , and M. Gullo . 2025. “Combining Microbial Cellulose With FeSO_4_ and FeCl_2_ by Ex Situ and In Situ Methods.” Polymers 17, no. 13: 1743. 10.3390/polym17131743. 40647754 PMC12251644

[crf370480-bib-0011] Barbosa, A. A. T. , H. G. S. de Araújo , P. N. Matos , M. A. G. Carnelossi , and A. A. de Castro . 2013. “Effects of Nisin‐Incorporated Films on the Microbiological and Physicochemical Quality of Minimally Processed Mangoes.” International Journal of Food Microbiology 164, no. 2–3: 135–140. 10.1016/j.ijfoodmicro.2013.04.004. 23673058

[crf370480-bib-0012] Batatinha, M. J. M. , M. M. S. Simas , M. B. Botura , T. C. Bitencourt , T. A. Reis , and B. Correa . 2007. “Fumonisins in Brewers Grain (Barley) Used as Dairy Cattle Feed in the State of Bahia, Brazil.” Food Control 18, no. 5: 608–612. 10.1016/j.foodcont.2006.02.008.

[crf370480-bib-0013] Benešová, K. , S. Běláková , R. Mikulíková , and Z. Svoboda . 2012. “Monitoring of Selected Aflatoxins in Brewing Materials and Beer by Liquid Chromatography/Mass Spectrometry.” Food Control 25, no. 2: 626–630. 10.1016/j.foodcont.2011.11.033.

[crf370480-bib-0014] Berenguer, C. V. , C. Andrade , J. A. M. Pereira , R. Perestrelo , and J. S. Câmara . 2023. “Current Challenges in the Sustainable Valorisation of Agri‐Food Wastes: A Review.” Processes 11, no. 1: 20. 10.3390/pr11010020.

[crf370480-bib-0015] Boudalia, S. , G. K. Symeon , V. Dotas , et al. 2026. “The Valorization of Agrifood Byproducts and Waste to Advance the Sustainable Development Goals: Current State and New Perspectives.” Sustainability 18, no. 5: 2165. 10.3390/su18052165.

[crf370480-bib-0016] Brachkova, M. I. , M. A. Duarte , and J. F. Pinto . 2010. “Preservation of Viability and Antibacterial Activity of *Lactobacillus* spp. in Calcium Alginate Beads.” European Journal of Pharmaceutical Sciences 41, no. 5: 589–596. 10.1016/j.ejps.2010.08.008. 20800681

[crf370480-bib-0017] Brugnoli, M. , S. La China , F. Lasagni , F. V. Romeo , A. Pulvirenti , and M. Gullo . 2023a. “Acetic Acid Bacteria in Agro‐Wastes: From Cheese Whey and Olive Mill Wastewater to Cellulose.” Applied Microbiology and Biotechnology 107, no. 11: 3729–3744. 10.1007/s00253-023-12539-8. 37115254

[crf370480-bib-0018] Brugnoli, M. , I. Mazzini , S. La China , L. De Vero , and M. Gullo . 2023b. “A Microbial Co‐Culturing System for Producing Cellulose‐Hyaluronic Acid Composites.” Microorganisms 11, no. 6: 1504. 10.3390/microorganisms11061504. 37375006 PMC10305311

[crf370480-bib-0019] Bukvicki, D. , M. D'Alessandro , S. Rossi , et al. 2023. “Essential Oils and Their Combination With Lactic Acid Bacteria and Bacteriocins to Improve the Safety and Shelf Life of Foods: A Review.” Foods 12, no. 17: 3288. 10.3390/foods12173288. 37685221 PMC10486891

[crf370480-bib-0020] Bukvicki, D. , L. Siroli , M. D'Alessandro , et al. 2020. “Unravelling the Potential of *Lactococcus lactis* Strains to be Used in Cheesemaking Production as Biocontrol Agents.” Foods 9, no. 12: 1815. 10.3390/foods9121815. 33297482 PMC7762361

[crf370480-bib-0021] Cardador, M. J. , M. Gallego , F. Prados , and J. Fernández‐Salguero . 2017. “Origin of Disinfection By‐Products in Cheese.” Food Additives & Contaminants: Part A 34, no. 6: 928–938. 10.1080/19440049.2017.1311421. 28346070

[crf370480-bib-0022] Carrera‐Rivera, A. , W. Ochoa , F. Larrinaga , and G. Lasa . 2022. “How to Conduct a Systematic Literature Review: A Quick Guide for Computer Science Research.” MethodsX 9: 101895. 10.1016/j.mex.2022.101895. 36405369 PMC9672331

[crf370480-bib-0023] Cé, N. , C. P. Noreña , and A. Brandelli . 2012. “Antimicrobial Activity of Chitosan Films Containing Nisin, Peptide P34, and Natamycin.” CyTA—Journal of Food 10, no. 1: 21–26. 10.1080/19476337.2010.537371.

[crf370480-bib-0024] Chen, F. , Z. Zhang , and J. Chen . 2018. “Infective Endocarditis Caused by *Lactococcus lactis* subsp. *lactis* and *Pediococcus pentosaceus*: A Case Report and Literature Review.” Medicine 97, no. 50: e13658. 10.1097/MD.0000000000013658. 30558065 PMC6320055

[crf370480-bib-0025] Cielecka, I. , M. Szustak , H. Kalinowska , et al. 2019. “Glycerol‐Plasticized Bacterial Nanocellulose‐Based Composites With Enhanced Flexibility and Liquid Sorption Capacity.” Cellulose 26, no. 9: 5409–5426. 10.1007/s10570-019-02501-1.

[crf370480-bib-0026] Cleenwerck, I. , P. De Vos , and L. De Vuyst . 2010. “Phylogeny and Differentiation of Species of the Genus *Gluconacetobacter* and Related Taxa Based on Multilocus Sequence Analyses of Housekeeping Genes and Reclassification of *Acetobacter xylinus* subsp. *Sucrofermentans* as *Gluconacetobacter Sucrofermentans* (Toyosaki et al. 1996) sp. nov., Comb. nov.” International Journal of Systematic and Evolutionary Microbiology 60, no. 10: 2277–2283. 10.1099/ijs.0.018465-0. 19915110

[crf370480-bib-0027] Concha‐Meyer, A. , R. Schöbitz , C. Brito , and R. Fuentes . 2011. “Lactic Acid Bacteria in an Alginate Film Inhibit *Listeria monocytogenes* Growth on Smoked Salmon.” Food Control 22, no. 3–4: 485–489. 10.1016/j.foodcont.2010.09.032.

[crf370480-bib-0028] Čuš, F. , H. B. Česnik , Š. V. Bolta , and A. Gregorčič . 2010. “Pesticide Residues in Grapes and During Vinification Process.” Food Control 21, no. 11: 1512–1518. 10.1016/j.foodcont.2010.04.024.

[crf370480-bib-0029] Dey, S. , M. Santra , M. Choudhury , A. R. Ghosh , and P. Samanta . 2025. “Food Waste Generation and Its Industrial Utilization: An Overview.” Environmental Science and Pollution Research 32, no. 12: 7493–7512. 10.1007/s11356-024-34252-3. 38990261

[crf370480-bib-0030] Dıblan, S. , and S. Kaya . 2018. “Antimicrobials Used in Active Packaging Films.” Food and Health 4, no. 1: 63–79. 10.3153/JFHS18007.

[crf370480-bib-0031] Dourado, F. , M. Gama , and A. C. Rodrigues . 2017. “A Review on the Toxicology and Dietetic Role of Bacterial Cellulose.” Toxicology Reports 4: 543–553. 10.1016/j.toxrep.2017.09.005. 29090119 PMC5655389

[crf370480-bib-0032] EFSA Panel on Biological Hazards (BIOHAZ) . Koutsoumanis, K. , A. Allende , et al. 2019. “Update of the List of QPS‐Recommended Biological Agents Intentionally Added to Food or Feed as Notified to EFSA 9: Suitability of Taxonomic Units Notified to EFSA Until September 2018.” EFSA Journal 17, no. 1: e05555. 10.2903/j.efsa.2019.5555. 32626100 PMC7328880

[crf370480-bib-0033] EFSA Panel on Biological Hazards (BIOHAZ) . Koutsoumanis, K. , A. Allende , et al. 2020. “Scientific Opinion on the Update of the List of QPS‐Recommended Biological Agents Intentionally Added to Food or Feed as Notified to EFSA (2017–2019).” EFSA Journal 18, no. 2: e05966. 10.2903/j.efsa.2020.5966. 32874212 PMC7448045

[crf370480-bib-0034] EFSA Panel on Biological Hazards (BIOHAZ) . Ricci, A. , A. Allende , et al. 2017. “Scientific Opinion on the Update of the List of QPS‐Recommended Biological Agents Intentionally Added to Food or Feed as Notified to EFSA.” EFSA Journal 15, no. 3: e04664. 10.2903/j.efsa.2017.4664. 32625421 PMC7010101

[crf370480-bib-0035] EFSA Panel on Food Additives and Nutrient Sources Added to Food (ANS) . Younes, M. , P. Aggett , et al. 2017. “Safety of Nisin (E 234) as a Food Additive in the Light of New Toxicological Data and the Proposed Extension of Use.” EFSA Journal 15, no. 12: e05063. 10.2903/j.efsa.2017.5063. 32625365 PMC7009836

[crf370480-bib-0036] European Commission, Joint Research Centre . 2024. Exploring the Environmental Performance of Alternative Food Packaging Products in the European Union: Life Cycle Impacts of Single Use and Multiple Use Packaging. Publications Office of the European Union. https://data.europa.eu/doi/10.2760/971274.

[crf370480-bib-0037] European Food Safety Authority (EFSA) . 2006. “Opinion of the Scientific Panel on Food Additives, Flavourings, Processing Aids and Materials in Contact With Food (AFC) Related to the Safety in Use of Nisin as a Food Additive in an Additional Category of Liquid Eggs.” EFSA Journal 4, no. 12: 314b. 10.2903/j.efsa.2006.314. PMC13130026 42079893

[crf370480-bib-0038] European Food Safety Authority (EFSA) . 2010. “EFSA Guidance for Those Carrying out Systematic Reviews. Application of Systematic Review Methodology to Food and Feed Safety Assessments to Support Decision Making.” EFSA Journal 8, no. 6: 1637. 10.2903/j.efsa.2010.1637. PMC13085193 42006960

[crf370480-bib-0039] European Food Safety Authority (EFSA) . 2021. “Administrative Guidance for the Preparation of Applications on Substances to be Used in Plastic Food Contact Materials.” EFSA Supporting Publications 18, no. 3: EN–6514. 10.2903/sp.efsa.2021.EN-6514.

[crf370480-bib-0040] European Food Safety Authority (EFSA) . Barthélémy, E. , C. Bolognesi , et al. 2023. “Principles That Could be Applicable to the Safety Assessment of the Use of Mixtures of Natural Origin to Manufacture Food Contact Materials.” EFSA Supporting Publications 20, no. 11: EN–8409. 10.2903/sp.efsa.2023.EN-8409.

[crf370480-bib-0041] Eurostat . 2023. “Packaging Waste Statistics.” https://ec.europa.eu/eurostat/statistics‐explained/index.php?title=Packaging_waste_statistics.

[crf370480-bib-0042] Fetting, C. 2020. The European Green Deal. ESDN.

[crf370480-bib-0043] García‐Bernet, D. , V. Ferraro , and R. Moscoviz . 2024. “Residues From the Food Industry: An Under‐Exploited Global Source of Biomolecules of Interest.” In Green Chemistry and Agro‐Food Industry: Towards a Sustainable Bioeconomy, edited by S. Baumberger , 155–190. Springer Nature Switzerland. 10.1007/978-3-031-54188-9_7.

[crf370480-bib-0044] Gerbaldo, G. A. , C. M. Pereyra , L. R. Cavaglieri , et al. 2011. “Surveillance of Aflatoxin and Microbiota Related to Brewer's Grain Destined for Swine Feed in Argentina.” Veterinary Medicine International 2011: 912480. 10.4061/2011/912480. 21547231 PMC3087608

[crf370480-bib-0045] Gialamas, H. , K. G. Zinoviadou , C. G. Biliaderis , and K. P. Koutsoumanis . 2010. “Development of a Novel Bioactive Packaging Based on the Incorporation of *Lactobacillus sakei* Into Sodium‐Caseinate Films for Controlling *Listeria monocytogenes* in Foods.” Food Research International 43, no. 10: 2402–2408. 10.1016/j.foodres.2010.09.020.

[crf370480-bib-0046] Girard, V. , J. Chaussé , and P. Vermette . 2024. “Bacterial Cellulose: A Comprehensive Review.” Journal of Applied Polymer Science 141, no. 15: e55163. 10.1002/app.55163.

[crf370480-bib-0047] Gonzalez Pereyra, M. L. , C. A. R. Rosa , A. M. Dalcero , and L. R. Cavaglieri . 2011. “Mycobiota and Mycotoxins in Malted Barley and Brewer's Spent Grain From Argentinean Breweries: Mycotoxins in Malt and Brewer's Grain.” Letters in Applied Microbiology 53, no. 6: 649–655. 10.1111/j.1472-765X.2011.03157.x. 21967240

[crf370480-bib-0048] Gottardi, D. , M. Ciccone , L. Siroli , R. Lanciotti , and F. Patrignani . 2022. “Use of *Yarrowia lipolytica* to Obtain Fish Waste Functional Hydrolysates Rich in Flavoring Compounds.” Fermentation 8, no. 12: 708. 10.3390/fermentation8120708.

[crf370480-bib-0049] Gottardi, D. , L. Siroli , G. Braschi , et al. 2023. “Selection of *Yarrowia lipolytica* Strains as Possible Solution to Valorize Untreated Cheese Whey.” Fermentation 9, no. 1: 51. 10.3390/fermentation9010051.

[crf370480-bib-0050] Gottardi, D. , L. Siroli , G. Braschi , et al. 2021. “High‐Pressure Homogenization and Biocontrol Agent as Innovative Approaches Increase Shelf Life and Functionality of Carrot Juice.” Foods 10, no. 12: 2998. 10.3390/foods10122998. 34945548 PMC8701166

[crf370480-bib-0051] Gromovykh, T. I. , A. G. Demchenko , E. G. Cheremnykh , et al. 2019. “Development of Bacterial Cellulose Biomaterial: Preparation and Establishment of Cytotoxicity for Eukaryotic Cells.” International Journal of Nanotechnology 16, no. 7/8/9/10/11/12: 354–365. https://www.inderscienceonline.com/doi/10.1504/IJNT.2019.102395.

[crf370480-bib-0052] Guillén, M. D. , P. Sopelana , and G. Palencia . 2004. “Polycyclic Aromatic Hydrocarbons and Olive Pomace Oil.” Journal of Agricultural and Food Chemistry 52, no. 7: 2123–2130. 10.1021/jf035259q. 15053562

[crf370480-bib-0053] Gullo, M. , S. La China , P. M. Falcone , and P. Giudici . 2018. “Biotechnological Production of Cellulose by Acetic Acid Bacteria: Current State and Perspectives.” Applied Microbiology and Biotechnology 102, no. 16: 6885–6898. 10.1007/s00253-018-9164-5. 29926141

[crf370480-bib-0054] Gullo, M. , A. Sola , G. Zanichelli , M. Montorsi , M. Messori , and P. Giudici . 2017. “Increased Production of Bacterial Cellulose as Starting Point for Scaled‐Up Applications.” Applied Microbiology and Biotechnology 101, no. 22: 8115–8127. 10.1007/s00253-017-8539-3. 28965208

[crf370480-bib-0055] Hack, M. , S. Nitz , and H. Parlar . 1997. “Behavior of [14C]Atrazine, [14C]Terbutylazine, and Their Major Metabolites in the Brewing Process.” Journal of Agricultural and Food Chemistry 45, no. 4: 1375–1380. 10.1021/jf9605411.

[crf370480-bib-0056] Haghighi, H. , M. Gullo , S. La China , et al. 2021. “Characterization of Bio‐Nanocomposite Films Based on Gelatin/Polyvinyl Alcohol Blend Reinforced With Bacterial Cellulose Nanowhiskers for Food Packaging Applications.” Food Hydrocolloids 113: 106454Ba.

[crf370480-bib-0057] Hakme, E. , I. Kallehauge Nielsen , J. Fermina Madsen , et al. 2024. “Fate of Pesticide Residues in Beer and Its By‐Products.” Food Additives & Contaminants: Part A 41, no. 1: 45–59. 10.1080/19440049.2023.2282557. 38039344

[crf370480-bib-0058] Haring, N. , B. Drábová , and M. Chňapek . 2026. “Circular Valorization of Brewer's Spent Grain and Brewer's Yeast: Pathways, Sustainability Implications, Techno‐Economic Feasibility, and Policy Perspectives.” Sustainability 18, no. 5: 2464. 10.3390/su18052464.

[crf370480-bib-0059] Huang, K. , C. Zhang , Y. Hu , M. Lacroix , and Y. Wang . 2025. “Holocellulose Nanofibrils as Effective Nisin Immobilization Substrates for Antimicrobial Food Packaging.” International Journal of Biological Macromolecules 304: 140741. 10.1016/j.ijbiomac.2025.140741. 39922342

[crf370480-bib-0060] Huang, Y.‐C. , D. Khumsupan , S.‐P. Lin , S. P. Santoso , H.‐Y. Hsu , and K. C. Cheng . 2024. “Production of Bacterial Cellulose (BC)/Nisin Composite With Enhanced Antibacterial and Mechanical Properties Through Co‐Cultivation of *Komagataeibacter xylinum* and *Lactococcus lactis* subsp. *lactis* .” International Journal of Biological Macromolecules 258: 128977. 10.1016/j.ijbiomac.2023.128977. 38154722

[crf370480-bib-0061] Infante‐Neta, A. A. , A. P. D'Almeida , and T. L. de Albuquerque . 2024. “Bacterial Cellulose in Food Packaging: A Bibliometric Analysis and Review of Sustainable Innovations and Prospects.” Processes 12, no. 9: 1975. 10.3390/pr12091975.

[crf370480-bib-0062] Jagannath, A. , P. S. Raju , and A. S. Bawa . 2010. “Comparative Evaluation of Bacterial Cellulose (Nata) as a Cryoprotectant and Carrier Support During the Freeze‐Drying Process of Probiotic Lactic Acid Bacteria.” LWT—Food Science and Technology 43, no. 8: 1197–1203. 10.1016/j.lwt.2010.03.009.

[crf370480-bib-0063] Junka, A. , K. Fijałkowski , A. Ząbek , et al. 2017. “Correlation Between Type of Alkali Rinsing, Cytotoxicity of Bio‐Nanocellulose and Presence of Metabolites Within Cellulose Membranes.” Carbohydrate Polymers 157: 371–379. 10.1016/j.carbpol.2016.10.007. 27987940

[crf370480-bib-0064] Kaboré, W. A. D. , R. Dembélé , T. S. Bagré , et al. 2018. “Characterization and Antimicrobial Susceptibility of *Lactococcus lactis* Isolated From Endodontic Infections in Ouagadougou, Burkina Faso.” Dentistry Journal 6, no. 4: 69. 10.3390/dj6040069. 30544668 PMC6313549

[crf370480-bib-0065] Kato, Y. , M. Kanayama , S. Yanai , H. Nozawa , O. Kanauchi , and S. Suzuki . 2018. “Safety Evaluation of Excessive Intake of *Lactococcus lactis* subsp. *lactis* JCM 5805: A Randomized, Double‐Blind, Placebo‐Controlled, Parallel‐Group Trial.” Food and Nutrition Sciences 9, no. 4: 401–416. 10.4236/fns.2018.94032.

[crf370480-bib-0066] Khelissa, S. , N.‐E. Chihib , and A. Gharsallaoui . 2021. “Conditions of Nisin Production by *Lactococcus lactis* subsp. *lactis* and Its Main Uses as a Food Preservative.” Archives of Microbiology 203, no. 2: 465–480. 10.1007/s00203-020-02054-z. 33001222

[crf370480-bib-0067] Kilemile, W. , K. E. Vulla , F. Mihafu , and V. Chandrasekaran . 2025. “Transforming Food Systems: A Review of Sustainable Approaches to Minimize Food Loss and Waste.” Food Science & Nutrition 13, no. 11: e71167. 10.1002/fsn3.71167. 41230386 PMC12603921

[crf370480-bib-0068] Ladakis, D. , H. Papapostolou , A. Vlysidis , and A. Koutinas . 2020. “Inventory of Food Processing Side Streams in European Union and Prospects for Biorefinery development.” In Food Industry Wastes, 2nd ed., edited by M. R. Kosseva and C. Webb , 181–199. Academic Press. 10.1016/B978-0-12-817121-9.00009-7.

[crf370480-bib-0069] Lasagni, F. , S. Cassanelli , and M. Gullo . 2024. “How Carbon Sources Drive Cellulose Synthesis in Two *Komagataeibacter xylinus* Strains.” Scientific Reports 14, no. 1: 20494. 10.3390/pr14030543. 39227724 PMC11371920

[crf370480-bib-0070] Lavasani, P. S. , E. Motevaseli , N. S. Sanikhani , and M. H. Modarressi . 2019. “ *Komagataeibacter xylinus* as a Novel Probiotic Candidate With High Glucose Conversion Rate Properties.” Heliyon 5, no. 4: e01571. 10.1016/j.heliyon.2019.e01571. 31183432 PMC6488717

[crf370480-bib-0071] Lee, J.‐Y. , S.‐E. Lee , and D.‐W. Lee . 2022. “Current Status and Future Prospects of Biological Routes to Bio‐Based Products Using Raw Materials, Wastes, and Residues as Renewable Resources.” Critical Reviews in Environmental Science and Technology 52, no. 14: 2453–2509. 10.1080/10643389.2021.1880259.

[crf370480-bib-0072] Li, Y. , H. Jiao , H. Zhang , et al. 2024. “Biosafety Consideration of Nanocellulose in Biomedical Applications: A Review.” International Journal of Biological Macromolecules 265: 130900. 10.1016/j.ijbiomac.2024.130900. 38499126

[crf370480-bib-0073] Li, Y. , Y. Wu , W. Quan , et al. 2021. “Quantitation of Furosine, Furfurals, and Advanced Glycation End Products in Milk Treated With Pasteurization and Sterilization Methods Applicable in China.” Food Research International 140: 110088. 10.1016/j.foodres.2020.110088. 33648304

[crf370480-bib-0074] Liang, Z.‐R. , H.‐I. Hsiao , and D. J. Jhang . 2020. “Synergistic Antibacterial Effect of Nisin, Ethylenediaminetetraacetic Acid, and Sulfite on Native Microflora of Fresh White Shrimp During Ice Storage.” Journal of Food Safety 40, no. 4: e12794. 10.1111/jfs.12794.

[crf370480-bib-0075] Ligarda‐Samanez, C. A. , M. L. Huamán‐Carrión , W. C. Calsina‐Ponce , et al. 2025. “Technological Innovations and Circular Economy in the Valorization of Agri‐Food By‐Products: Advances, Challenges and Perspectives.” Foods 14, no. 11: 1950. 10.3390/foods14111950. 40509479 PMC12155285

[crf370480-bib-0147] Lin, H. , L. Ni , H. Chen , and W. Xu . 2022. “A simple and versatile strategy for sensitive SIDA‐UHPLC‐MS/MS analysis of Alternaria toxins in olive oil.” Analytica Chimica Acta, 1232, 340451. 10.1016/j.aca.2022.340451. 36257757

[crf370480-bib-0076] Liu, J. , M. Bacher , T. Rosenau , S. Willför , and A. Mihranyan . 2018. “Potentially Immunogenic Contaminants in Wood‐Based and Bacterial Nanocellulose: Assessment of Endotoxin and (1,3)‐β‐D‐Glucan Levels.” Biomacromolecules 19, no. 1: 150–157. 10.1021/acs.biomac.7b01334. 29182312

[crf370480-bib-0077] Lopes, P. , M. M. C. Sobral , G. R. Lopes , et al. 2023. “Mycotoxins' Prevalence in Food Industry by‐products: A Systematic Review.” Toxins 15, no. 4: 249. 10.3390/toxins15040249. 37104187 PMC10142126

[crf370480-bib-0078] Marcos Muntal, B. , M. Aymerich Calvet , M. Garriga Turón , and J. Arnau . 2013. “Active Packaging Containing Nisin and High Pressure Processing as Post‐Processing Listericidal Treatments for Convenience Fermented Sausages.” Food Control 30, no. 1: 325–330. 10.1016/j.foodcont.2012.07.019.

[crf370480-bib-0079] Maresca, D. , and G. Mauriello . 2022. “Development of Antimicrobial Cellulose Nanofiber‐based Films Activated With Nisin for Food Packaging Applications.” Foods 11, no. 19: 3051. 10.3390/foods11193051. 36230127 PMC9564163

[crf370480-bib-0080] McCarthy, W. P. , T. F. O'Callaghan , M. Danahar , et al. 2018. “Chlorate and Other Oxychlorine Contaminants Within the Dairy Supply Chain.” Comprehensive Reviews in Food Science and Food Safety 17, no. 6: 1561–1575. 10.1111/1541-4337.12393. 33350140

[crf370480-bib-0081] Mehdizadeh, M. , M. A. K. Abed , D. K. Al‐Taey , Z. Abideen , S. Morya , and T. Chameh . 2026. “Valorization of Agricultural Waste Into Biodegradable Nanocomposites for Sustainable Food Packaging: A Systematic Review.” Circular Economy and Sustainability 6, no. 2: 25.7. 10.1007/s43615-026-00746-0.

[crf370480-bib-0082] Millette, M. , W. Smoragiewicz , and M. Lacroix . 2004. “Antimicrobial Potential of Immobilized *Lactococcus lactis* subsp. *lactis* ATCC 11454 Against Selected Bacteria.” Journal of Food Protection 67, no. 6: 1184–1189. 10.4315/0362-028X-67.6.1184. 15222547

[crf370480-bib-0083] Monaci, L. , R. Pilolli , L. Quintieri , L. Caputo , A. Luparelli , and E. De Angelis . 2024. “Casein: Allergenicity and Molecular properties.” In Casein, edited by M. El‐Bakry and B. M. Mehta , 363–382. Academic Press. 10.1016/B978-0-443-15836-0.00008-1.

[crf370480-bib-0084] Moreda, W. , R. Rodríguez‐Acuña , M. del Carmen Pérez‐Camino , and A. Cert . 2004. “Determination of High Molecular Mass Polycyclic Aromatic Hydrocarbons in Refined Olive Pomace and Other Vegetable Oils.” Journal of the Science of Food and Agriculture 84, no. 13: 1759–1764. 10.1002/jsfa.1877.

[crf370480-bib-0085] Mossburger, J. , and K. A. Scherf . 2024. “Gluten Migration From Biodegradable Food Contact Materials Poses a Risk to Celiac Disease Patients.” European Food Research and Technology 250, no. 11: 2711–2718. 10.1007/s00217-024-04570-4.

[crf370480-bib-0086] Musiejuk, M. , and P. Kafarski . 2023. “Engineering of Nisin as a Means for Improvement of Its Pharmacological Properties: A Review.” Pharmaceuticals 16, no. 8: 1058. 10.3390/ph16081058. 37630973 PMC10459688

[crf370480-bib-0087] Napavichayanun, S. , S. Ampawong , T. Harnsilpong , A. Angspatt , and P. Aramwit . 2018. “Inflammatory Reaction, Clinical Efficacy, and Safety of Bacterial Cellulose Wound Dressing Containing Silk Sericin and Polyhexamethylene Biguanide for Wound Treatment.” Archives of Dermatological Research 310, no. 10: 795–805. 10.1007/s00403-018-1871-3. 30302557

[crf370480-bib-0088] Navarro, S. , G. Pérez , N. Vela , L. Mena , and G. Navarro . 2005. “Behavior of Myclobutanil, Propiconazole, and Nuarimol Residues During Lager Beer Brewing.” Journal of Agricultural and Food Chemistry 53, no. 22: 8572–8579. 10.1021/jf0505832. 16248555

[crf370480-bib-0089] Navarro, S. , N. Vela , and G. Navarro . 2011. “Fate of Triazole Fungicide Residues During Malting, Mashing and Boiling Stages of Beer Making.” Food Chemistry 124, no. 1: 261–270. 10.1016/j.foodchem.2010.06.033.

[crf370480-bib-0090] Njieukam, J. A. , M. Ciccone , D. Gottardi , et al. 2024. “Microbiological, Functional, and Chemico‐Physical Characterization of Artisanal Kombucha: An Interesting Reservoir of Microbial Diversity.” Foods 13, no. 12: 191947. 10.3390/foods13121947. PMC11202501 38928888

[crf370480-bib-0091] Novickij, V. , R. Stanevičienė , G. Staigvila , et al. 2020. “Effects of Pulsed Electric Fields and Mild Thermal Treatment on Antimicrobial Efficacy of Nisin‐Loaded Pectin Nanoparticles for Food Preservation.” LWT 120: 108915. 10.1016/j.lwt.2019.108915.

[crf370480-bib-0092] O'Connor, A. M. , G. L. Lovei , J. Eales , et al. 2012. “Implementation of Systematic Reviews in EFSA Scientific Outputs Workflow.” EFSA Supporting Publications 9, no. 12: EN–367. 10.2903/sp.efsa.2012.EN-367.

[crf370480-bib-0093] Olczyk, M. , and M. Kuc‐Czarnecka . 2025. “European Green Deal Index: A New Composite Tool for Monitoring European Union's Green Deal Strategy.” Journal of Cleaner Production 495: 145077. 10.1016/j.jclepro.2025.145077.

[crf370480-bib-0094] Oyewole, O. A. , A. Izuafa , and I. A. Ayomide . 2026. “Microbial‐Derived Bioactive Metabolites as Next‐Generation Solutions for Sustainable Food Preservation and Safety.” Applied Research 5, no. 2: e70080. 10.1002/appl.70080.

[crf370480-bib-0095] Page, M. J. , J. E. McKenzie , P. M. Bossuyt , et al. 2021. “Updating Guidance for Reporting Systematic Reviews: Development of the PRISMA 2020 Statement.” Journal of Clinical Epidemiology 134: 103–112. 10.1016/j.jclinepi.2021.02.003. 33577987

[crf370480-bib-0096] Panda, J. , A. K. Mishra , Y. K. Mohanta , K. Patowary , P. R. Rauta , and B. Mishra . 2024. “Exploring Biopolymer for Food and Pharmaceuticals Application in the Circular Bioeconomy: An Agro‐Food Waste‐to‐Wealth Approach.” Waste and Biomass Valorization 15, no. 10: 5607–5637. 10.1007/s12649-024-02452-0.

[crf370480-bib-0097] Pandey, A. , A. Singh , and M. K. Singh . 2025. “Biocellulose.” In The Handbook of Paper‐Based Sensors and Devices: Volume 1: Materials and Technologies, edited by G. Korotcenkov , 237–269. Springer Nature Switzerland. 10.1007/978-3-031-91080-7_10.

[crf370480-bib-0098] Panghal, A. , S. Janghu , K. Virkar , Y. Gat , V. Kumar , and N. Chhikara . 2018. “Potential Non‐Dairy Probiotic Products—A Healthy Approach.” Food Bioscience 21: 80–89. 10.1016/j.fbio.2017.12.003.

[crf370480-bib-0099] Papaioannou, E. H. , R. Mazzei , F. Bazzarelli , et al. 2022. “Agri‐Food Industry Waste as Resource of Chemicals: The Role of Membrane Technology in Their Sustainable Recycling.” Sustainability 14, no. 3: 1483. 10.3390/su14031483.

[crf370480-bib-0100] Parfitt, J. , K. Luyckx , D. Jarosz , et al. 2018. “EU Policy Review for Food Waste Prevention and Valorisation [Report].” https://www.ecologic.eu/15826.

[crf370480-bib-0101] Patrignani, F. , L. Siroli , F. Gardini , and R. Lanciotti . 2016. “Contribution of Two Different Packaging Material to Microbial Contamination of Peaches: Implications in Their Microbiological Quality.” Frontiers in Microbiology 7: 938. 10.3389/fmicb.2016.00938. 27379067 PMC4909747

[crf370480-bib-0102] Pereira, R. N. , R. M. Rodrigues , Ó. L. Ramos , F. Xavier Malcata , J. A. Teixeira , and A. A. Vicente . 2016. “Production of Whey Protein‐Based Aggregates Under Ohmic Heating.” Food and Bioprocess Technology 9, no. 4: 576–587. 10.1007/s11947-015-1651-4.

[crf370480-bib-0103] Pérez‐Lucas, G. , G. Navarro , and S. Navarro . 2023. “Comprehensive Review on Monitoring, Behavior, and Impact of Pesticide Residues During Beer‐Making.” Journal of Agricultural and Food Chemistry 71, no. 4: 1820–1836. 10.1021/acs.jafc.2c07830. 36651341 PMC9896562

[crf370480-bib-0104] Piacentini, K. C. , L. O. Rocha , G. D. Savi , L. Carnielli‐Queiroz , L. De Carvalho Fontes , and B. Correa . 2019. “Assessment of Toxigenic *Fusarium* Species and Their Mycotoxins in Brewing Barley Grains.” Toxins 11, no. 1: 1. 10.3390/toxins11010031. PMC6357013 30634556

[crf370480-bib-0105] Pietri, A. , A. Mulazzi , G. Piva , and T. Bertuzzi . 2016. “Fate of Aflatoxin M1 During Production and Storage of Parmesan Cheese.” Food Control 60: 478–483. 10.1016/j.foodcont.2015.08.032.

[crf370480-bib-0106] Pigaleva, M. A. , M. V. Bulat , T. I. Gromovykh , et al. 2019. “A New Approach to Purification of Bacterial Cellulose Membranes: What Happens to Bacteria in Supercritical Media?” The Journal of Supercritical Fluids 147: 59–69. 10.1016/j.supflu.2019.02.009.

[crf370480-bib-0107] Pinto, F. C. M. , A. C. A. X. De‐Oliveira , R. R. De‐Carvalho , et al. 2016. “Acute Toxicity, Cytotoxicity, Genotoxicity and Antigenotoxic Effects of a Cellulosic Exopolysaccharide Obtained From Sugarcane Molasses.” Carbohydrate Polymers 137: 556–560. 10.1016/j.carbpol.2015.10.071. 26686163

[crf370480-bib-0108] Pinto, M. S. , A. F. de Carvalho , A. C. dos Santos Pires , et al. 2011. “The Effects of Nisin on *Staphylococcus aureus* Count and the Physicochemical Properties of Traditional Minas Serro Cheese.” International Dairy Journal 21, no. 2: 90–96. 10.1016/j.idairyj.2010.08.001.

[crf370480-bib-0109] Pires, P. N. , E. A. Vargas , M. B. Gomes , et al. 2019. “Aflatoxins and Ochratoxin A: Occurrence and Contamination Levels in Cocoa Beans From Brazil.” Food Additives & Contaminants: Part A 36, no. 5: 815–824. 10.1080/19440049.2019.1600749. 30973077

[crf370480-bib-0110] Rao, M. , A. Bast , and A. de Boer . 2021. “Valorized Food Processing By‐Products in the EU: Finding the Balance Between Safety, Nutrition, and Sustainability.” Sustainability 13, no. 8: 4428. 10.3390/su13084428.

[crf370480-bib-0111] Regulation (EC) No 1935/2004 of the European Parliament and of the Council of 27 October 2004 on materials and articles intended to come into contact with food and repealing Directives 80/590/EEC and 89/109/EEC . *Official Journal of the European Union*, L 338, 13.11.2004, 4–1. https://eur‐lex.europa.eu/eli/reg/2004/1935/oj/eng.

[crf370480-bib-0112] Ribeiro, E. , and A. Alves . 2008. “Comparative Study of Screening Methodologies for Ochratoxin A Detection in Winery By‐Products.” Analytical and Bioanalytical Chemistry 391, no. 4: 1443–1450. 10.1007/s00216-008-1861-y. 18256811

[crf370480-bib-0113] Romão, S. , A. Bettencourt , and I. A. C. Ribeiro . 2022. “Novel Features of Cellulose‐Based Films as Sustainable Alternatives for Food Packaging.” Polymers 14, no. 22: 4998. 10.3390/polym14224968. 36433095 PMC9699531

[crf370480-bib-0114] Rossi, S. , D. Gottardi , L. Siroli , et al. 2024. “Functional and Biochemical Characterization of Pre‐Fermented Ingredients Obtained by the Fermentation of Durum Wheat by‐products.” Journal of Functional Foods 116: 106136. 10.1016/j.jff.2024.106136.

[crf370480-bib-0115] Schmidt, L. , O. D. Prestes , P. R. Augusti , and J. C. Fonseca Moreira . 2023. “Phenolic Compounds and Contaminants in Olive Oil and Pomace: A Narrative Review of Their Biological and Toxic Effects.” Food Bioscience 53: 102626. 10.1016/j.fbio.2023.102626.

[crf370480-bib-0116] Shahmohammadi Jebel, F. , and H. Almasi . 2016. “Morphological, Physical, Antimicrobial and Release Properties of ZnO Nanoparticles‐Loaded Bacterial Cellulose Films.” Carbohydrate Polymers 149: 8–19. 10.1016/j.carbpol.2016.04.089. 27261725

[crf370480-bib-0117] Sharma, A. , S. Ashique , M. Kaushik , et al. 2026. “Polysaccharides and Carbohydrate Polymers: Innovations From Nature to Industry.” Journal of the Science of Food and Agriculture. 10.1002/jsfa.70541. PMC13442779 41760377

[crf370480-bib-0118] Sharma, B. K. , A. Jha , D. Bhalani , et al. 2025. “Advancements in Bio‐Resource‐Based Polymers and Composites: Sustainable Alternatives to Non‐Biodegradable Plastics for a Greener Future: A Review.” Current Green Chemistry 12, no. 2: 82219. 10.2174/0122133461372269250503082219.

[crf370480-bib-0119] Sharma, H. B. , K. R. Vanapalli , V. S. Cheela , et al. 2020. “Challenges, Opportunities, and Innovations for Effective Solid Waste Management During and Post COVID‐19 Pandemic.” Resources, Conservation and Recycling 162: 105052. 10.1016/j.resconrec.2020.105052. 32834486 PMC7362850

[crf370480-bib-0120] Sharma, V. , M.‐L. Tsai , P. Nargotra , et al. 2022. “Agro‐Industrial Food Waste as a Low‐Cost Substrate for Sustainable Production of Industrial Enzymes: A Critical Review.” Catalysts 12, no. 11: 1373. 10.3390/catal12111373.

[crf370480-bib-0121] Shelver, W. L. , S. J. Lupton , N. W. Shappell , D. J. Smith , and H. Hakk . 2018. “Distribution of Chemical Residues Among Fat, Skim, Curd, Whey, and Protein Fractions in Fortified, Pasteurized Milk.” ACS Omega 3, no. 8: 8697–8708. 10.1021/acsomega.8b00762. 31459001 PMC6645336

[crf370480-bib-0122] Shimizu, A. , R. Hase , D. Suzuki , et al. 2019. “ *Lactococcus lactis* Cholangitis and Bacteremia Identified by MALDI‐TOF Mass Spectrometry: A Case Report and Review of the Literature on *Lactococcus lactis* Infection.” Journal of Infection and Chemotherapy 25, no. 2: 141–146. 10.1016/j.jiac.2018.07.010. 30100399

[crf370480-bib-0123] Signorello, L. , M. P. Arena , M. Brugnoli , F. V. Romeo , and M. Gullo . 2025. “Valorization of Olive Mill Wastewater by Selective Sequential Fermentation.” Foods 14, no. 13: 2170. 10.3390/foods14132170. 40646922 PMC12249083

[crf370480-bib-0124] Signorello, L. , M. Brugnoli , M. P. Arena , and M. Gullo . 2026. “Isolation and Reassembly of Cultivable Bacteria and Yeasts for Kombucha Tea Fermentation.” Fermentation 12, no. 2: 100. 10.3390/fermentation12020100.

[crf370480-bib-0125] Sinha, S. 2024. “An Overview of Biopolymer‐Derived Packaging Material.” Polymers from Renewable Resources 15, no. 2: 193–209. 10.1177/20412479241226884.

[crf370480-bib-0126] Siroli, L. , L. Camprini , M. B. Pisano , F. Patrignani , and R. Lanciotti . 2019. “Volatile Molecule Profiles and Anti‐*Listeria monocytogenes* Activity of Nisin Producers *Lactococcus lactis* Strains in Vegetable Drinks.” Frontiers in Microbiology 10: 563. 10.3389/fmicb.2019.00563. 30972045 PMC6443959

[crf370480-bib-0127] Siroli, L. , B. Giordani , S. Rossi , et al. 2022. “Antioxidant and Functional Features of Pre‐Fermented Ingredients Obtained by the Fermentation of Milling By‐Products.” Fermentation 8, no. 12: 722. 10.3390/fermentation8120722.

[crf370480-bib-0128] Siroli, L. , V. Glicerina , F. Capelli , et al. 2023. “Influence of High‐Pressure Homogenization Treatments Combined With Lysozyme‐Activated Packaging on Microbiological and Technological Quality of Vegetable Smoothie During Shelf Life.” Food Packaging and Shelf Life 37: 101093. 10.1016/j.fpsl.2023.101093.

[crf370480-bib-0129] Siroli, L. , F. Patrignani , M. D'Alessandro , E. Salvetti , S. Torriani , and R. Lanciotti . 2020. “Suitability of the Nisin Z‐Producer *Lactococcus lactis* subsp. *lactis* CBM 21 to be Used as an Adjunct Culture for Squacquerone Cheese Production.” Animals 10, no. 5: 782. 10.3390/ani10050782. 32365951 PMC7277329

[crf370480-bib-0130] Siroli, L. , F. Patrignani , D. I. Serrazanetti , et al. 2017. “Survival of Spoilage and Pathogenic Microorganisms on Cardboard and Plastic Packaging Materials.” Frontiers in Microbiology 8: 2606. 10.3389/fmicb.2017.02606. 29312271 PMC5743701

[crf370480-bib-0131] Siroli, L. , F. Patrignani , D. I. Serrazanetti , et al. 2016. “Use of a Nisin‐Producing *Lactococcus lactis* Strain, Combined With Natural Antimicrobials, to Improve the Safety and Shelf‐Life of Minimally Processed Sliced Apples.” Food Microbiology 54: 11–19. 10.1016/j.fm.2014.11.008 25583340

[crf370480-bib-0132] Sumini, M. , G. J. S. Andrade , C. A. Tischer , R. K. T. Kobayashi , and G. Nakazato . 2025. “Production of Bacterial Cellulose by *Komagataeibacter xylinus*: Biochemistry, Synthesis and Applications.” Cellulose 32, no. 1: 81–94. 10.1007/s10570-024-06179-y.

[crf370480-bib-0133] Townsend, K. , J. Laffan , and G. Hayman . 2021. “Carboxymethylcellulose Excipient Allergy: A Case Report.” Journal of Medical Case Reports 15, no. 1: 565. 10.1186/s13256-021-03180-y. 34819140 PMC8611968

[crf370480-bib-0134] Tsermoula, P. , B. Khakimov , J. H. Nielsen , and S. B. Engelsen . 2021. “WHEY—The Waste‐Stream That Became More Valuable Than the Food Product.” Trends in Food Science & Technology 118: 230–241. 10.1016/j.tifs.2021.08.025.

[crf370480-bib-0135] Uribe‐Velázquez, T. , C. E. Najar‐Almanzor , F. R. Osuna‐Orozco , et al. 2026. “Cheese Whey Valorization via Microbial Fermentation (Lactic Acid Bacteria, Yeasts/Fungi, and Microalgae), Postbiotic Production, and Whey‐Based Encapsulation Strategies.” Fermentation 12, no. 1: 42. 10.3390/fermentation12010042.

[crf370480-bib-0136] U.S. Food and Drug Administration . 1992. CFR—Code of Federal Regulations Title 21. U.S. Food and Drug Administration. https://www.accessdata.fda.gov/scripts/cdrh/cfdocs/cfcfr/cfrsearch.cfm?fr=184.1538.

[crf370480-bib-0137] Wang, H. , Y. Niu , J. Pan , Q. Li , and R. Lu . 2020. “Antibacterial Effects of *Lactobacillus acidophilus* Surface‐Layer Protein in Combination With Nisin Against *Staphylococcus aureus* .” LWT 124: 109208. 10.1016/j.lwt.2020.109208.

[crf370480-bib-0138] Wang, X. , W. Kang , J. Li , Z. Deng , and J. Gao . 2025. “Nisin: Harnessing Nature's Preservative for the Future of Food Safety and Beyond.” Critical Reviews in Food Science and Nutrition 65, no. 34: 9045–9070. 10.1080/10408398.2025.2517822. 40531107

[crf370480-bib-0139] Waqas, M. , S. Z. Iqbal , A. F. Abdull Razis , et al. 2021. “Occurrence of Aflatoxins in Edible Vegetable Seeds and Oil Samples Available in Pakistani Retail Markets and Estimation of Dietary Intake in Consumers.” International Journal of Environmental Research and Public Health 18, no. 15: 8015. 10.3390/ijerph18158015. 34360308 PMC8345775

[crf370480-bib-0140] Xie, F. , W. Hu , X. Meng , C. Hu , X. Li , and H. Chen . 2024. “A Comparison of the Physicochemical Properties, Allergenicity, and Stability of Recombinant β‐Lactoglobulin: Development of Standard Bovine Milk Allergens.” Food Bioscience 61: 104956. 10.1016/j.fbio.2024.104956.

[crf370480-bib-0141] Xing, W. , W. Liu , B. Yang , et al. 2026. “In Situ Multi‐Crosslinked Bacterial Cellulose With Enhanced Mechanical and Barrier Properties for Antimicrobial Food Contact Packaging.” International Journal of Biological Macromolecules 344, no. 1: 150546. 10.1016/j.ijbiomac.2026.150546. 41605394

[crf370480-bib-0142] Yoon, S. H. , and G. B. Kim . 2022. “Inhibition of *Listeria monocytogenes* in Fresh Cheese Using a Bacteriocin‐Producing *Lactococcus lactis* CAU2013 Strain.” Food Science of Animal Resources 42, no. 6: 1009. 10.5851/kosfa.2022.e48. 36415575 PMC9647177

[crf370480-bib-0143] Younis, H. G. , F. Abdelrahman , and H. R. Abdellatif . 2026. “Recent Applications and Future Prospects of Biopolymer‐Based Composites in the Food Industry.” In Biopolymers: Green and Sustainable Approaches for Drug Delivery, Food Products and Packaging, 363–423. Springer. 10.1007/978-981-95-2258-3.

[crf370480-bib-0144] Zhao, H. , F. Zhang , J. Chai , and J. Wang . 2020. “Effect of Lactic Acid Bacteria on *Listeria monocytogenes* Infection and Innate Immunity in Rabbits.” Czech Journal of Animal Science 65, no. 1: 23–30. 10.17221/247/2019-CJAS.

[crf370480-bib-0145] Zhao, Z. , A. A. Kohansal , Y. Liu , Y. Huang , H. Xiao , and F. Seidi . 2026. “Antimicrobial Bacterial Cellulose: Fabrication Strategies and Antimicrobial Efficiencies.” Cellulose 33, no. 4: 1781–1831. 10.1007/s10570-026-06969-6.

[crf370480-bib-0146] Zotta, T. , L. Solieri , L. Iacumin , C. Picozzi , and M. Gullo . 2020. “Valorization of Cheese Whey Using Microbial Fermentations.” Applied Microbiology and Biotechnology 104, no. 7: 2749–2764. 10.1007/s00253-020-10408-2. 32009200

